# Acid blue 40 dye decolorization using magnetite nanoparticles with reduced graphene oxide and mesoporous silica as Fenton catalysts

**DOI:** 10.1038/s41598-025-91382-5

**Published:** 2025-03-14

**Authors:** Nady Fathy, Khadiga Abas, Amina Attia, Mona Shouman

**Affiliations:** Physical Chemistry Department, Advanced Materials Technology and Mineral Resources Research Institute, National Research Centre, 33 El Buhouth St., Dokki, P.O. 12622, Cairo, Egypt

**Keywords:** Rice husk ash, SBA-16, Reduced graphene oxide, Fe_3_O_4_, Fenton-like heterogeneous catalysis, Chemistry, Materials science

## Abstract

Synthetic dyes are predominantly emitted into the eco-environment resulting, in harmful effects on the environment and human. This study presents a new perspective on the mesoporous silica (SBA-16) and reduced graphene oxide (rGO) obtained from rice husk ash as substrates for Fe_3_O_4_ nanoparticles (NPs) to investigate their morphological and Fenton catalytic characteristics towards degradation of synthetic acid blue 40 dye (AB40). The adsorption and Fenton catalytic properties of AB40 dye by the prepared Fe_3_O_4_, Fe_3_O_4_/SBA-16 and Fe_3_O_4_/rGO catalysts were examined. The successful synthesis of such catalysts was affirmed by the results obtained from FE-SEM, EDX, TEM, FTIR, XRD and nitrogen adsorption measurements. The adsorption of AB40 dye followed the Langmuir model, with maximum adsorption capacities of 169.2, 21.1 and 16.6 mg/g for Fe_3_O_4_, for Fe_3_O_4_/SBA-16 and Fe_3_O_4_/rGO, respectively. This result was explained based on their specific surface areas. The decolorization efficiency was estimated through several factors, including initial dye concentration, pH and H_2_O_2_ concentration. The results disclosed that a catalyst dose = 1 g/L, initial dye concentration = 50 mg/L, pH = 3 and [H_2_O_2_] = 15 mmol/L are the optimum conditions for full decolorization of AB40 within 60 min at 35 °C. The prepared Fe_3_O_4_ NPs exhibited a superior Fenton activity at 25 °C and pH 3. However, both composites increased Fenton performance above 25 °C, indicating that SBA-16 and rGO substrates can enhance the stability of Fe^2+^ to generate a higher amount of hydroxyl radicals. Regeneration results disclosed that the obtained Fenton-like catalysts revealed notably high catalytic efficiency (> 95%) and stability, with minimal decrease in activity observed after running four cycles of AB40 dye degradation at pH 3 and 35 °C. Thus, this study demonstrated that both SBA-16 and rGO substrates obtained from rice husk ash improved the reusability and stability of Fe_3_O_4_ catalysts in wastewater treatment using heterogeneous Fenton reactions.

## Introduction

Homogeneous and heterogeneous Fenton-like oxidation technologies, among other advanced oxidation processes (AOPs)^1,2^ have been successfully employed for the decolorization of organic effluents based on their strong oxidation of Fe^2+^-based catalysts to generate hydroxyl radicals^3^. However, the heterogeneous Fenton-like oxidation process is a more efficient technology for wastewater treatment since it overwhelms the disadvantages associated with homogeneous oxidation, such as a narrow working pH range, large quantity of iron sludge and low H_2_O_2_ utilization efficiency^4,5^. This reaction has been widely recognized and extensively researched as a potent and promising approach for environmental remediation^5^. To date, ferrous (Fe^2+^)-based catalysts have received increasing attention due to their earth abundance, good biocompatibility, comparatively low toxicity, ready availability and environmentally benign nature, as well as the high natural abundance of iron element in the earth’s crust^5,6^. Among them, magnetite (Fe_3_O_4_)-based iron oxide catalysts can be easily synthesized by practical and straightforward methods. The circulation of Fe(III)/Fe(II) active sites and surface reactions of catalysts play significant roles in enhancing the generation rates of hydroxyl radicals during Fenton-like reactions^5^. Popular methods such as solvothermal and hydrothermal processes, thermal decomposition, microemulsion processes, and co-precipitation procedures are widely used for the synthesis of iron-based compounds^7^.

Fe_3_O_4_ nanoparticles (NPs) have attracted significant attention due to their strong electrical resistivity, distinctive magnetic characteristics, biocompatibility, and excellent chemical stability^5,6^. However, using Fe_3_O_4_ NPs in Fenton-like reactions presents drawbacks. These include operation at a limited pH (> 4), and when the pH of the solution rises, the majority of the Fe_3_O_4_ NPs agglomerate and then tend to decrease their total surface area and hinder reactant passage. Hence, this would lower the Fenton catalytic performance and catalyst’s regeneration efficiency^7^. To overcome these limitations, the loading and fixation of Fe_3_O_4_ NPs on porous supports such as silica, zeolites, metal–organic frameworks, activated carbons, carbon nanotubes and graphene oxides for heterogeneous Fenton-like oxidation of organic pollutants have been recently performed^4,7–14^.

In this context, constructing a heterogeneous-Fenton reaction with high chemical and mechanical stability and active catalysts is a promising alternative to prevail above issues that associated with the homogeneous-Fenton reactions. Notably, the inclusion of magnetic nanoparticles into porous substrates allows convenient and economical magnetic separation from the liquid phase instead of centrifugation and filtration steps. Mesoporous silica materials, including MCM 41, HMS, KIT, SBA, and others, have drawn a lot of interest as substrates due to their easily adjustable surface, application-dependent tunable porosity, and inertness^15–18^. It has been reported that the functionalization of SBA-15 with Fe_3_O_4_ NPs exhibits remarkable catalytic activity in the synthesis of dihydropyrano pyrazole derivatives^15^. This can be linked to the strong basic sites in the guanidine group, the Lewis acid site present in Fe_3_O_4_, and the high surface area achieved for SBA-15 with Fe_3_O_4_^15^. The potential of Fe_3_O_4_ NPs immobilized on SBA-15 mesoporous silica as Fenton and photo-Fenton catalysts toward the degradation of sulfamethoxazole antibiotic and orange II dye under visible light irradiation was studied, showing a good reusability for 10 cycles^16^. Another report indicated that the immobilization of Fe_3_O_4_@SBA-15 as a photocatalyst exhibited significantly high catalytic activity and stability toward the degradation of malachite green dye for five cycles^17^. Au nanoparticles functionalized with melamine-α–chloroacetic acid groups supported by Fe_3_O_4_/SBA-16 were found to serve as highly effective, heterogeneous, and recyclable catalysts for the reduction of organic dyes such as methylene blue and methyl orange dyes in aqueous solutions^18^. For comparison, SBA-16 is more porous than SBA-15, allowing the organic functional groups of the catalyst to easily fit within its pores, giving an excellent catalytic performance and superior adsorption capacity^18,19^.

On the other hand, reduced graphene oxide (rGO) is a highly carbonaceous oxygenated material with hydrophilic functional groups such as hydroxyl and epoxide groups on the basal planes and carbonyl and carboxyl groups at the sheet edges^20^. This material is produced through the chemical oxidation of synthetic or natural graphite powder^20^. Accordingly, magnetic graphene oxide functionalized by Fe_3_O_4_ materials has gained a considerable interest as a heterogeneous catalyst for the Fenton-like degradation of dye pollutants (e.g., acid orange 7, methylene blue, acid blue 113, etc.)^21–25^. For example, Zubir et al.^23^ prepared graphene oxide-iron oxide (GO-Fe_3_O_4_) nanocomposites by co-precipitating iron salts onto GO sheets in a basic solution. The catalyst exhibited greater degradation ability of acid orange 7 than that of individual Fe_3_O_4_ NPs in a heterogeneous Fenton-like reaction. This was due to the GO substrate, which attained high dispersibility and chemical stability of Fe_3_O_4_ NPs and increased the total surface area^23^. Therefore, this enhancement in catalytic activity was attributed to the synergistic effects between both GO sheets and Fe_3_O_4_ NPs.

**Fig. 1:**
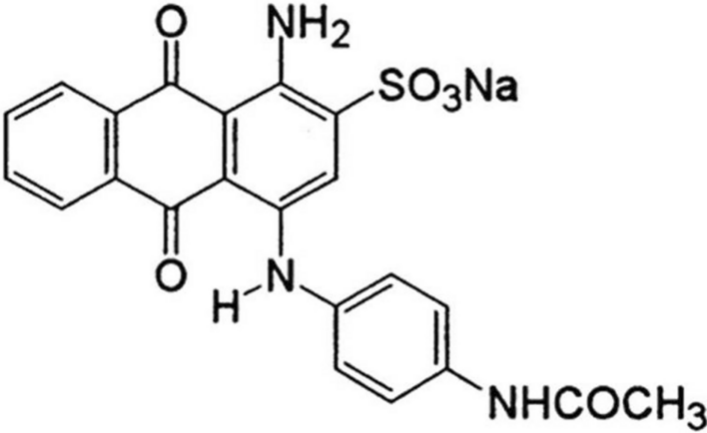
Molecular structure of acid blue 40 dye.

Acid dyes are widely used in the textile and apparel sectors for coloring wool, nylon, cotton, and silk materials^26–30^. Despite its advantageous use, it might result in side effects such as skin irritation and stomach, intestinal, and eye burns^26^. Additionally, the presence of dyes in rivers and other bodies of surface water can harm the aquatic ecology and thus raise the water’s hardness and turbidity^27^. Regarding to their environmental hazards, especially acid blue 40 (AB40) dye, the elimination of AB40 from wastewater has attracted much interest in previous studies^26–30^. AB40 dye is a member of the second-most significant class of anthraquinone dyes and its molecular structure is depicted in Fig. 1^28^.

When comparing the preparation of SBA-16 and rGO materials from low-cost precursors to their counterparts made from expensive precursors such as tetraethyl orthosilicate and graphite, respectively, these materials are thought to be extremely inexpensive as Fe_3_O_4_ catalyst supports. Specifically, rice husk ash is a significant agricultural waste containing silica and carbon sources. The utilization of such waste to produce SBA-16 and rGO as Fe_3_O_4_-based substrates is an extremely significant strategy^19,20^. To the best of our knowledge, Fenton-like decolorization studies of AB40 dye using Fe_3_O_4_/SBA-16 and Fe_3_O_4_/rGO have not been reported yet. Therefore, the main objective of the current study is to examine novel and low-cost substrates such as SBA-16 and rGO, prepared from both silicate and carbon precursors of rice husk ash as reported in our works^19,20^. The Fe_3_O_4_/SBA-16 and Fe_3_O_4_/rGO composites were synthesized by immobilizing Fe_3_O_4_ NPs on SBA-16 and rGO sheets during the co-precipitation method of Fe(II) and Fe(III) salts in an alkaline pH. The fabricated samples were extensively characterized using field emission-scanning electron microscopy (FE-SEM), energy dispersive X-ray (EDX), transmission electron microscopy (TEM), Fourier transform infrared (FTIR) spectroscopy, X-ray diffraction (XRD), and nitrogen sorption to better explain their structures and functionalities. Finally, the catalytic activity of the prepared samples in the degradation of AB40 dye was evaluated in a heterogeneous Fenton-like reaction under different conditions.

## Materials and methods

Mesoporous silica (SBA-16) and reduced graphene oxide (rGO) were prepared in the laboratory from rice husk ash, as mentioned in our previous works^19,20^. Other chemicals were of analytical grade and utilized directly without additional purification. Ferric chloride (FeCl_3_∙6H_2_O, 98.5%), ferrous chloride (FeCl_2_∙4H_2_O, 98%), ammonium hydroxide (NH_4_OH, 25%), hydrochloric acid (HCl, 36%), and ethylene glycol (EG, 95%) were purchased from Merck. Hydrogen peroxide (H_2_O_2_, 35%) was provided by Fisher Scientific Co. Acid blue 40 dye (AB40) was supplied by Sigma-Aldrich.

### Preparation of magnetic Fe_3_O_4_ NPs

An aqueous suspension of magnetic nanoparticles was prepared by co-precipitation of Fe(III) and Fe(II) in ethylene glycol with NH_4_OH at 80 °C, as described by Silva et al.^31^, with minor changes. In brief, ferric chloride hexahydrate (FeCl_3_.6H_2_O) (1.38 g) and ferrous chloride tetrahydrate (FeCl_2_.4H_2_O) (0.8 g) were dissolved in 40 mL of ethylene glycol (concentration ratio Fe^3+^:Fe^2+^  = 1.5:1). After 1 h of ultrasonication, an additional 60 mL of ethylene glycol was added while the mixture was vigorously stirred. The precipitating agent, a 10 mL NH_4_OH solution, is added dropwise until pH ~ 9 to avoid the coagulation and large particle size precipitation. The mixture was heated at 80 °C for 2 h under reflux. After cooling, the produced precipitate was centrifuged at 3000 rpm for 15 min to separate a black precipitate, cleaned with deionized water and finally with ethanol, then dried in an air oven at 60 °C for 24 h.

The magnetite precipitate is formed according to the following reaction^31^:

1 $${\text{Fe}}^{2 + } + 2{\text{Fe}}^{3 + } + 8{\text{OH}}^{ - } \to {\text{Fe}}_{3} {\text{O}}_{4} + 4{\text{H}}_{2} {\text{O}}$$

### Preparation of Fenton‐like magnetic catalysts

The as-prepared SBA-16 was obtained hydrothermally at 100 °C for 24 h^19^. While, the rGO sample was fabricated from activated carbon of rice husk ash as previously reported in elsewhere^19,20^. Both products were used as supports for the prepared catalysts. Each support (0.4 g) was sonicated separately in 50 mL of ethylene glycol for 1 h. In a typical procedure for in situ co-precipitation of magnetite nanoparticles, 0.69 g of Fe (III) and 0.4 g of Fe (II) were dissolved in another 50 mL of ethylene glycol, followed by sonication for 10 min, and then poured over the supports dispersed in ethylene glycol solution under vigorous stirring at 80 °C. After that, 10 mL of NH_4_OH solution was added dropwise until the mixture’s pH reached 9. The reaction was maintained under reflux at 80 °C for 2 h. The produced catalysts were centrifuged, washed, and dried overnight in an air oven at 60 °C. The resulting samples were nominated as Fe_3_O_4_/SBA-16 and Fe_3_O_4_/rGO. To measure the surface pH of the prepared samples, 20 mg of each sample was conducted in 20 mL distilled water and warmed at 40 °C for 20 min. Upon cooling, a digital pH meter (pH-HANNA-1010) was used to measure the pH.

### Physicochemical characterization

Field emission-scanning electron microscopy (FE-SEM) combined with an energy dispersive X-ray (EDX) analyzer (QUANTA FEG 250E-SEM, Japan) was implemented to inspect the surface morphology and elemental composition of the generated magnetite and magnetite-based composites. A transmission electron microscope (TEM, JEOL/JEM-1230) was used to explore the particle size and morphology of Fe_3_O_4_ samples. The functional surface groups of the obtained samples were inspected employing the KBr pellet approach on a Fourier Transform Infrared (FTIR, NICOLET 8700 spectrometer, Thermo Scientific, United Kingdom) in the spectral range of 400–4000 cm^−1^ with four resolutions averaged over 40 scans. X-ray diffraction diffractometer (XRD, Bruker D8 Advance, Germany) with a CuKα1 target and a monochromator set at 40 kV and 40 mA was used to determine the crystal phases of the produced samples. The specific surface area using the Brunauer–Emmett–Teller (BET) method and the pore characteristics of the synthesized samples were assessed by adsorption–desorption of N_2_ at − 196 °C using (MicrotracBEL Corp., Belsorp Version 6.3.2.1).

### Batch adsorption study of AB40 dye

The treated dye solution containing the obtained catalysts was magnetically stirred without H_2_O_2_ at 25 °C for 24 h in order to attain adsorption–desorption equilibrium between the dye solution and the catalyst. This was done to compare the percentage removal (%) of AB40 dye by either adsorption or the Fenton oxidation process. Stock solutions of dissolved AB40 dye (10–300 mg/L) were prepared. The working pH of the dye solution was 7.6. Batch adsorption experiments were conducted by shaking 10 mL of each dye concentration with 10 mg of the catalyst in a rotating shaker at 220 rpm for 24 h at 25 °C. The equilibrium adsorption amount, q_e_ (mg/g), was determined using the following Eq. (2):

2 $${\text{q}}_{\text{e}} =\frac{\text{V }\left({\text{C}}_{\text{o}}-{\text{C}}_{\text{e}}\right)}{\text{m}}$$

where C_o_ and C_e_ (mg/L) are the initial and equilibrium concentrations in the liquid phase, V (mL) the volume of the equilibrium solution, and m (mg) is the mass of the adsorbent. The removal efficiency (% R) of the AB40 dye was used to establish the percentage of dye removal as follows:

3 $$\text{R }(\text{\%}) =\frac{ ({\text{C}}_{\text{o}}-{\text{C}}_{\text{e}})}{{\text{C}}_{\text{o}}}$$

To calculate the monolayer adsorption capacity of the obtained samples, Langmuir isotherm^32^ was applied and symbolized by the linear-form Eq. (4):

4 $$\frac{{\text{C}}_{\text{e}}}{{\text{q}}_{\text{e}}}=\frac{1}{{\text{K}}_{\text{L}}{.\text{q}}_{\text{m}}}+ \frac{{\text{C}}_{\text{e}}}{{\text{q}}_{\text{m}}}$$

Assuming C_e_ is the equilibrium concentration of dye (mg/L), q_e_ is the equilibrium adsorption capacity (mg/g), q_m_ is the monolayer adsorption capacity (mg/g), and K_L_ is the Langmuir constant (L/mg) that specifies the adsorption behavior. The equilibrium parameter R_L_, referred to as the separation factor, is a dimensionless constant^32^ and can be expressed using the following Eq. (5), where C_o_ is the starting concentration at the highest removal efficiency.

5 $${\text{R}}_{\text{L}} =\frac{1}{\left(1 + {\text{K}}_{\text{L}}.{\text{C}}_{\text{o}}\right)}$$

The R_L_ value determines if the adsorption feature is irreversible when R_L_ = 0, favorable when 0 < R_L_ < 1, or linear if R_L_ = 1^32^.

By assuming the adsorption characteristics for the heterogeneous surface, Freundlich isotherm was used and formulated as the following Eq. (6)^33^:

6 $${\text{log q}}_{{\text{e}}} = {\text{log K}}_{{\text{F}}} + \frac{1}{{\text{n}}}{\text{log C}}_{{\text{e}}}$$

where K_F_ is the Freundlich isotherm constant and n is the adsorption intensity constant. The separation of the two phases at n = 1 is concentration-independent. If 1/n is less than 1, typical adsorption is predicted. On the contrary, 1/n > 1 indicates reciprocal adsorption^33^.

### Fenton oxidation experiments

The Fenton degradation of AB40 dye was carried out in 50 mL glass bottles under various experimental settings, including different dye concentrations (50–300 mg/L), dye solution pH (3–6), reaction temperature (25–45 °C), and oxidant dose [H_2_O_2_] (15–60 mmol/L). For this purpose, 10 mg of the produced catalyst was added to 10 mL of AB40 aqueous solution with stirring for 1 h without adding H_2_O_2_. To adjust the pH, either 0.1 M HCl or 0.1 M NaOH solution was used. Before and after each oxidative degradation experiment, the dye solution’s absorbance was measured and recorded. The UV–Vis spectrum of each AB40 solution was determined using a model UV-2401PC spectrophotometer with a 1 cm quartz cuvette at 620 nm, the maximum absorbance wavelength of the dye solution. The AB40 degradation (%) was computed by Eq. (7) below, where A_o_ and A_t_ indicate the initial concentration of AB40 and at time (t), respectively^34^.

7 $${\text{Decolorization}}\,\% = \frac{{{\text{A}}_{{\text{o}}} - {\text{A}}_{{\text{t}}} }}{{{\text{A}}_{{\text{o}}} }} \times 100$$

The Fenton reaction was started upon adding H_2_O_2_, whereas the process was stopped by adding 0.5 mL of 10 M NaOH^34^.

### Decolorization kinetics

To illustrate how the hydroxyl radicals (HO^·^) oxidize the tested organic dye, the decolorization kinetic rates of AB40 dye (C_o_ = 100 mg/L) by the Fenton process were investigated at 35 °C at various contact times ranging from 2 min to 1 h. Considering that the AB40 dye decolorization in water follows pseudo-first-order reaction kinetics^34^, as shown in the following Eq. (8).

8 $$\ln \left( {\frac{{\text{A}}}{{{\text{A}}_{{\text{o}}} }}} \right) = - {\text{K}}_{{\text{d}}} {\text{t}}$$

The slope of the logarithmic plot of color absorbance (A/A_°_) vs. time (t) was employed to figure out the decolorization rate constant (k_d_). In addition, the pseudo-second-order model proposed by Ho and McKay^35^ was also tested, as shown in the Eq. (9):

9 $$\frac{{\text{t}}}{{{\text{A}}_{{\text{t}}} }} = \frac{1}{{{\text{K}}_{2} {\text{A}}_{2}^{{\text{e}}} }} + \frac{{\text{t}}}{{{\text{A}}_{{\text{e}}} }}$$

where, A_e_ is the equilibrium absorbance of the dye solution. The pseudo-second-order rate constant (K_2_) was considered from the plot of t/A_t_ versus t. Absorbance readings of remaining AB40 dye solutions were recorded at triplicate, with a relative standard deviation of < 3%.

## Results and discussion

### Morphology and elemental compositions of samples

**Fig. 2:**
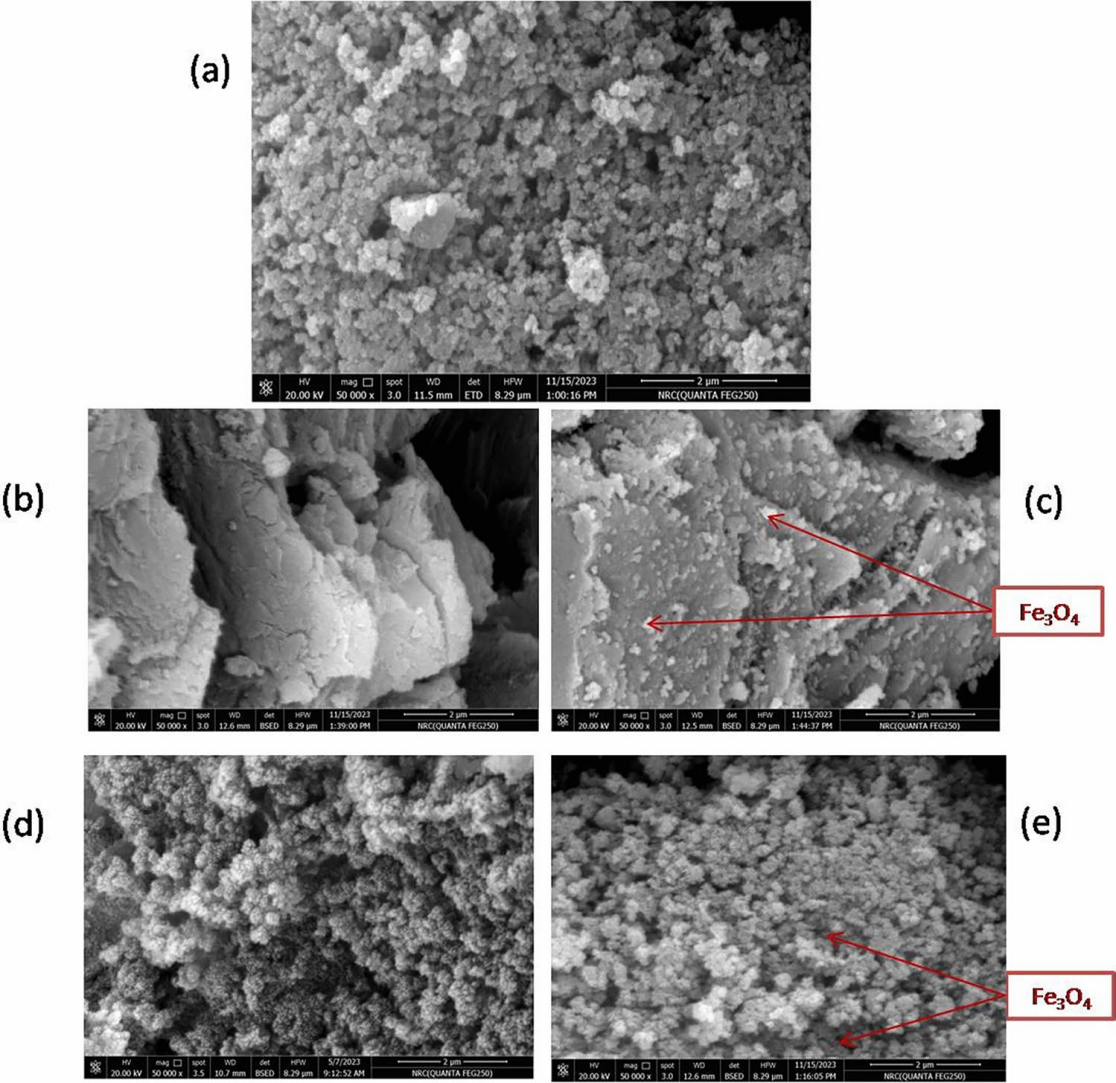
FE-SEM images of (**a**) Fe_3_O_4_ NPs, (**b**) rGO, (**c**) Fe_3_O_4_/rGO, (**d**) SBA-16 and (**e**) Fe_3_O_4_/SBA-16.

As observed in Fig. 2a–e, FE-SEM images demonstrate the surface morphology of the as-prepared catalysts. Figure 2a reveals that the surface of Fe_3_O_4_ NPs is composed of aggregated spherical-like particles with a relatively non-uniform distribution. In a previous study, it was explained that using NH_4_OH as a precipitating agent during the co-precipitation of Fe_3_O_4_ NPs resulted in spherical-Fe_3_O_4_ NPs due to the nucleation of Fe(OH)_2_ and FeOOH^31^. As seen in Fig. 2b, the obtained rGO sheets reveal irregularly stacked and thick platelets of graphene with irregular edges, wrinkled surfaces, and folding sheets as a result of the reduction of graphene sheets. Upon loading Fe_3_O_4_ NPs on rGO substrate, as shown in Fig. 2c, Fe_3_O_4_ NPs are apparently distributed between the rGO sheets, confirming the successful loading of Fe_3_O_4_ NPs on rGO. The SEM image of SBA-16 (Fig. 2d) also aggregated spherical-like particles. For Fe_3_O_4_/SBA-16, the distribution of spherical Fe_3_O_4_ NPs on the SBA-16, a mesoporous substrate, can also be detected in Fig. 2e. Earlier, similar results observed for SBA-15/ Fe_3_O_4_ by Hassanzadeh‑Afruzi et al.^15^.

**Fig. 3:**
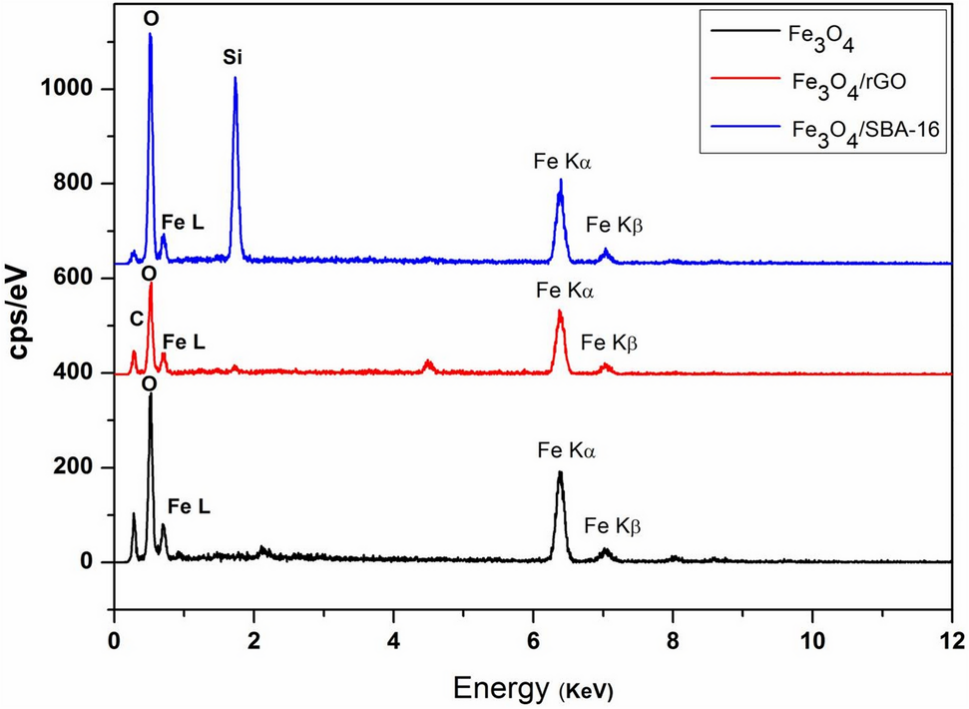
EDX spectra of the as-prepared samples.

**Fig. 4:**
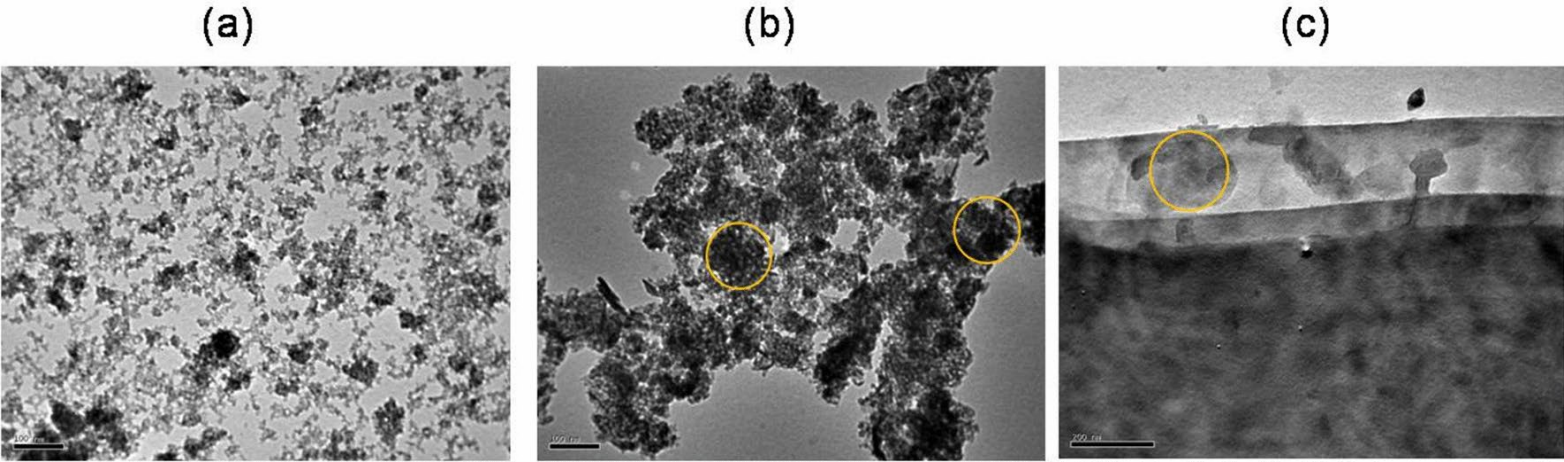
TEM images of (**a**) Fe_3_O_4_, (**b**) Fe_3_O_4_/SBA-16, and (**c**) Fe_3_O_4_/rGO at 100 nm and 200 nm scales, respectively (yellow circles indicate Fe_3_O_4_ distribution).

Figure 3 reveals the EDX spectra analysis to explore the elemental compositions in the as-prepared samples. The spectra detect distinctive peaks of Fe, O, C and Si that are related to the elemental compositions in Fe_3_O_4_, Fe_3_O_4_/rGO and Fe_3_O_4_/SBA-16, respectively, confirming the successful incorporation of Fe_3_O_4_ within rGO and SBA-16 substrates. In addition, Fig. 4 depicts TEM images of the prepared samples. Fe_3_O_4_ spherical-like nanoparticles composed of smaller grains with an average particle size of 4–10 nm were formed as shown in Fig. 4a, resulting in large total surface area. The yellow circles in TEM images (Fig. 4b, c) showed that Fe_3_O_4_ NPs are dispersed throughout the porous matrix of SBA-16 particles and graphene layers of rGO.

### Surface functional groups analysis

**Fig. 5:**
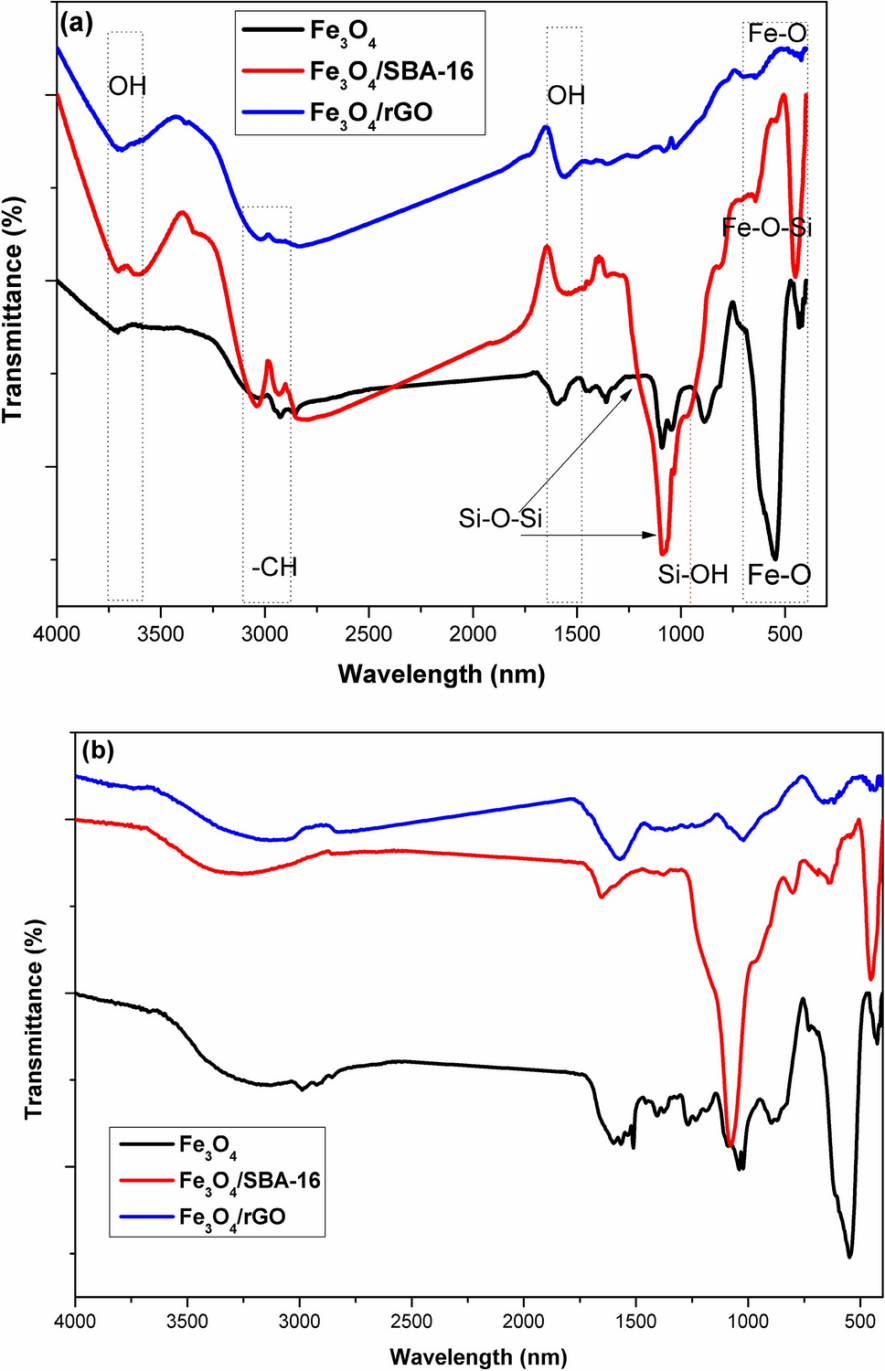
FTIR spectra of prepared samples (**a**) before and (**b**) after Fenton reaction.

FTIR spectroscopy is appropriately utilized to investigate absorption bands of magnetic composite systems containing SBA-16 and rGO (Fe_3_O_4_, Fe_3_O_4_/SBA-16, and Fe_3_O_4_/rGO) in the current investigation. The FTIR spectra of the prepared catalysts before and after dye decolorization are shown in Fig. 5a, b. The spectra of all prepared catalysts prior to the Fenton reaction show a deep peak at wavelength 550 cm^−1^, which is related to the stretching vibrations of Fe–O bonds of Fe_3_O_4_ nanoparticles^31^. This demonstrates that the magnetic core is present and more prominent in the magnetite nanoparticles, whereas the distinctive bands of the maghemite phase (Fe_2_O_3_) in the spectral region 740–620 cm^−1^ are not detected^15,31^. This outcome affirms that the co-precipitation using NH_4_OH permits the synthesis of a single magnetite phase. Small shoulder bands at 800 and 965 cm^−1^ are ascribed to the existence of symmetric stretching bands of Si–O–Si and Si–OH, respectively, along with the development of an asymmetric band of Si–O at 1085 cm^−1^^15–19^. Remarkably, the band at 640 cm^−1^ is the distinctive band of the magnetite/silica bond, Fe–O–Si^17^, demonstrating that the silica/magnetite composite has been successfully formed. For Fe_3_O_4_/rGO composite, the bands at 1018 and 1360 cm^−1^ are distinctive to the C–O and C–OH bonds, which are active sites for bonding with Fe atoms, respectively^21–25^. A characteristic band frequency at 1567 cm^−1^ in the produced Fe_3_O_4_/rGO FTIR spectra is attributed to C=C stretching vibrations on the rGO surface, while the band around 2925 cm^−1^ is assigned to the stretching vibration of –CH_2_ group^11,12^. Additionally, the band at approximately 1600 cm^−1^ and the broad band centered at 3350 cm^−1^ are attributed to OH-bending and OH-stretching, respectively^20^.

In order to comprehend the diversity in surface groups of the samples obtained following the Fenton dye degradation process, the FTIR spectra of samples are shown in Fig. 5b. Results reveal that the band intensities remarkably decreased or increased without corresponding variations in their location. Given that AB40 is an anthraquinone dye with two oxygen and two nitrogen atoms, which may function as ligands to complex with Fe^+2^ and Fe^+3^ sites on the Fe-based powder surface. This observation implies that chemisorption may contribute to the decolorization process followed by the decomposition of dye molecules^21,22^. A wide band at a wavelength of 3250 cm^−1^ is described to the presence of amino groups from chemisorbed dye, produced during the breakdown of dye upon catalyzing hydrogen peroxide to OH^·^ radicals by iron salt^7^.

### Structural analysis

**Fig. 6:**
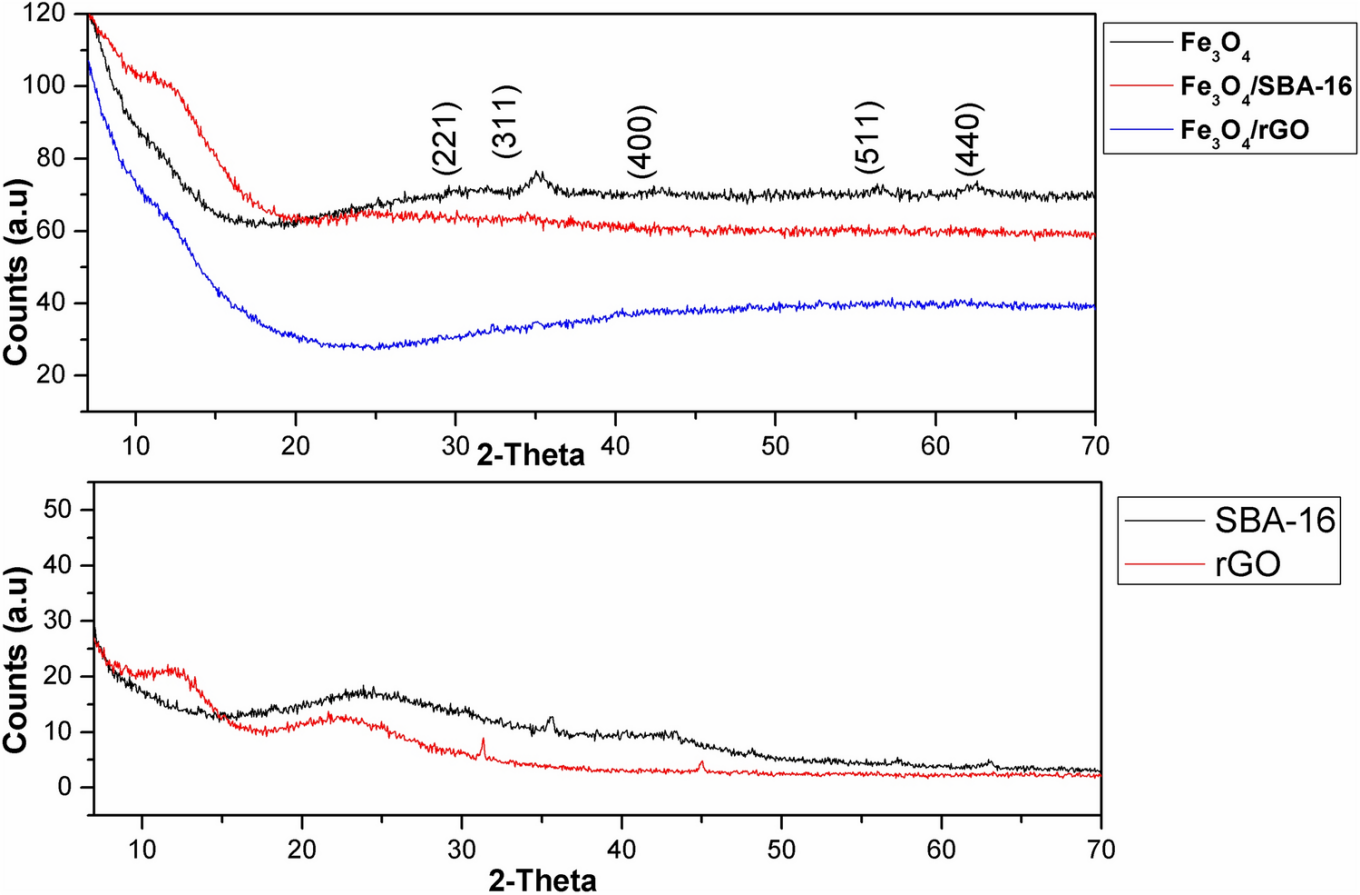
XRD patterns of the prepared samples.

X-ray diffraction patterns of the magnetite nanoparticles and their prepared composite catalysts are displayed in Fig. 6. The diffraction peaks for magnetite are consistent with the standard patterns for Fe_3_O_4_ at 2Ө = 31°, 35°, 42.8°, 56.4°, and 62.5° according to JCPDS # 08-4611^22^. For the prepared composite with SBA-16, no diffraction peak is observed except for a broad amorphous band centered at 23°, which is the characteristic peak for amorphous SiO_2_^19^. For rGO, the XRD pattern often showed a large diffraction peak at around 26.5° (d = 0.31 nm) that corresponded to the C(002) reflection, indicating the partially ordered structure in rGO which established from π-π re-stacking after reduction. Moreover, many sp^3^ carbon atoms established during the oxidation process of graphite could lead to the formation of amorphous structure in the Fe_3_O_4_/rGO composite^20,36^. Consequently, the crystallinity of Fe_3_O_4_ was reduced upon mixing with SBA-16 and rGO to form magnetite composites, confirming the successful preparation of magnetic composites.

### Surface textural analysis

**Fig. 7:**
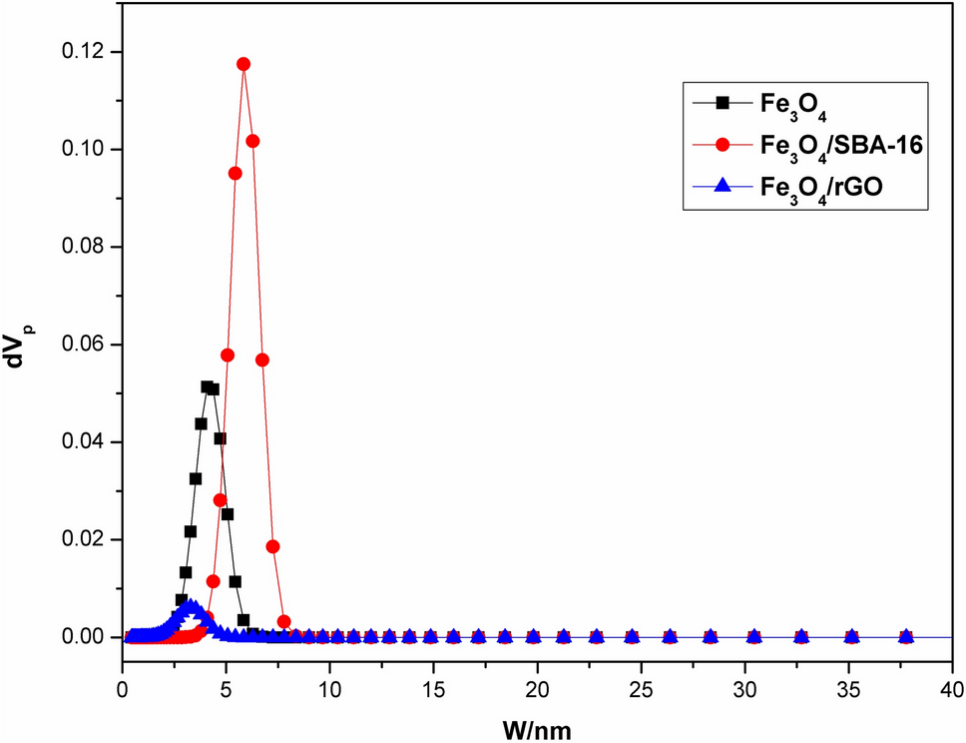
NLDFT pore size distribution analysis of prepared based samples.

**Fig. 8:**
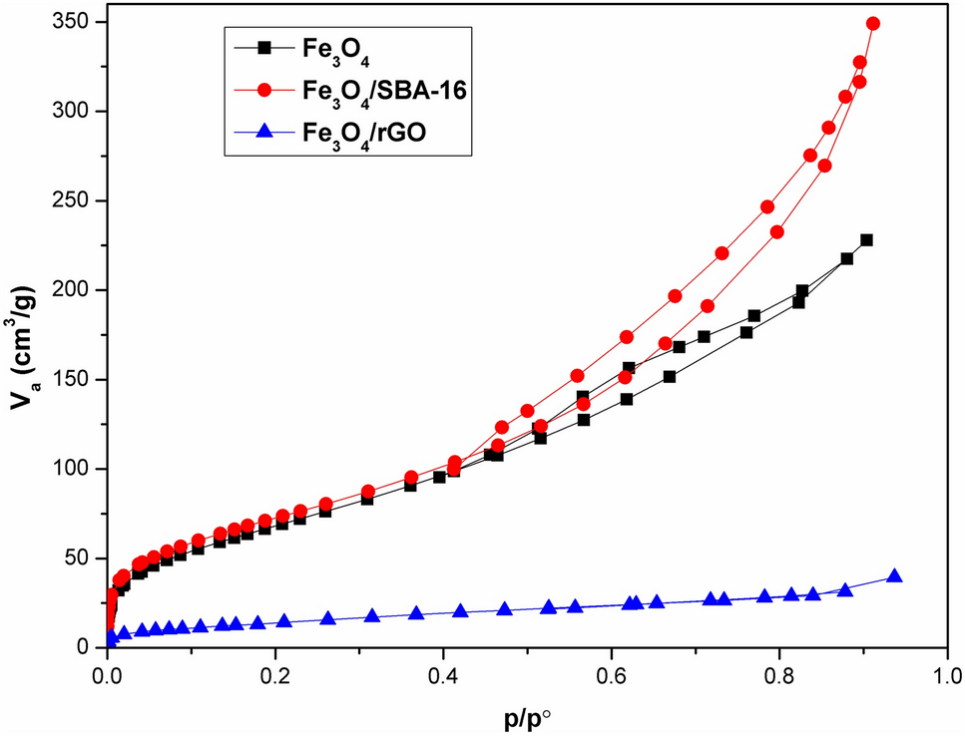
N_2_ adsorption–desorption measurements of prepared samples.

By measuring the N_2_ adsorption–desorption isotherms at 77 K, the surface area and textural features of the SBA-16 and rGO coated with in-situ generated magnetite particles were examined. The results are illustrated in Figs. 7 and 8, as well as Table 1. Based on the IUPAC category and pore size distribution (PSD) patterns (Fig. 7), the adsorbents developed in this investigation may be classified into mesoporous groups^13^. The results demonstrated in Fig. 8 suggest that all samples exhibited the properties of type IV with an H2 hysteresis loop, which is characteristic of ordered mesoporous structures accompanied by high regularity. The textural parameters obtained in Table 1 show that Fe_3_O_4_/rGO and Fe_3_O_4_/SBA-16 composites have specific surface areas lower than that of Fe_3_O_4_ NPs. Previous studies^19,20^ reported that the textural parameters of SBA-16 and rGO were 415 and 195 m^2^/g which were decreased after loading with Fe_3_O_4_ NPs to be ~ 276 and 55 m^2^/g for Fe_3_O_4_/SBA-16 and Fe_3_O_4_/rGO respectively, as seen in Table 1. Such a decrease in the total surface areas and total pore volumes may be attributed to the aggregation of Fe_3_O_4_ NPs on the surfaces of both SBA-16 and rGO samples^8^. In contrast to our findings, Hua et al.^24^ found that mixing Fe_3_O_4_ NPs with GO in presence of gallic acid significantly increased the Fenton catalytic performance of Fe_3_O_4_/GO for decolorizing methylene blue. They concluded that gallic acid increased the specific surface area and expedited electron transport, resulting in the rapid regeneration of Fe(II) species to enhance the creation of HO^·^ radicals. The total surface areas of Fe_3_O_4_ and Fe_3_O_4_/gallic acid/GO were 93.18 m^2^/g and 226.19 m^2^/g, respectively^24^.

**Table 1:** Surface area and pore size distribution of prepared catalysts.

Specimens	pH_PZC_	S_BET_ (m^2^/g)	Average pore diameter (nm)	V_total_ (cm^3^/g)
Fe_3_O_4_	3.5	267.5	5.3	0.35
Fe_3_O_4_/SBA-16	7.7	275.7	7.8	0.54
Fe_3_O_4_/rGO	4.7	54.8	4.5	0.06

### Determination of pH-point of zero charge

Magnetite, classified as an amphoteric solid, has the ability to acquire both positive and negative charges through the processes of protonation that represented by the reaction (FeOH + H^+^ → FeOH^2+^) and deprotonation that indicated by (FeOH + H^+^ → FeO^−^ + H^+^)^37^. These reactions take place at the FeOH sites that are formed on the surface of magnetite when it is dispersed in water. It is therefore essential to ascertain the point of zero charge (pHpzc) of the prepared Fe_3_O_4_ NPs in order to predict the nature of the charge on their surface at a specific pH. Results of pH_PZC_ for the samples are shown in Fig. S1 and Table 1. The prepared Fe_3_O_4_ and Fe_3_O_4_/rGO catalysts showed an acidic surface nature due to pH_PZC_ are 3.5 and 4.7, respectively. Whereas Fe_3_O_4_/SBA-16 has pH_PZC_ = 7.7 indicating a neutral surface is obtained. The surfaces of Fe_3_O_4_/SBA-16 and Fe_3_O_4_/rGO are predominantly adorned with Fe–O, hydroxyl, carboxyl, epoxy, and silanol functional groups, which exhibit variations in their forms depending on the pH level. Below the pHpzc, the surface of the adsorbent carries a positive charge, facilitating the adsorption of AB40 dye anions. Negatively charged anions of AB40 dye are more likely to be adsorbed at acidic pHs because there are more positively charged adsorbent sites and fewer negatively charged ones resulting in electrostatic attractions^26–30,37^. Additionally, there exists competition between hydroxide ions (OH^−^) at elevated pH levels and dye anions for the positively charged adsorption sites, lowering the amount adsorbed of dye. Hence, it can be concluded that the surface properties of Fe_3_O_4_ exhibit high total surface area and more active acidic sites showing high magnetic properties that can be adsorbed the protonated AB40 dye molecules in acidic media. However, these acidic sites over the fabricated magnetic composites decreased upon increasing value of pH_PZC_ leading to a decrease in the adsorption of AB40 dye.

### Effect of initial dye concentration

**Fig. 9:**
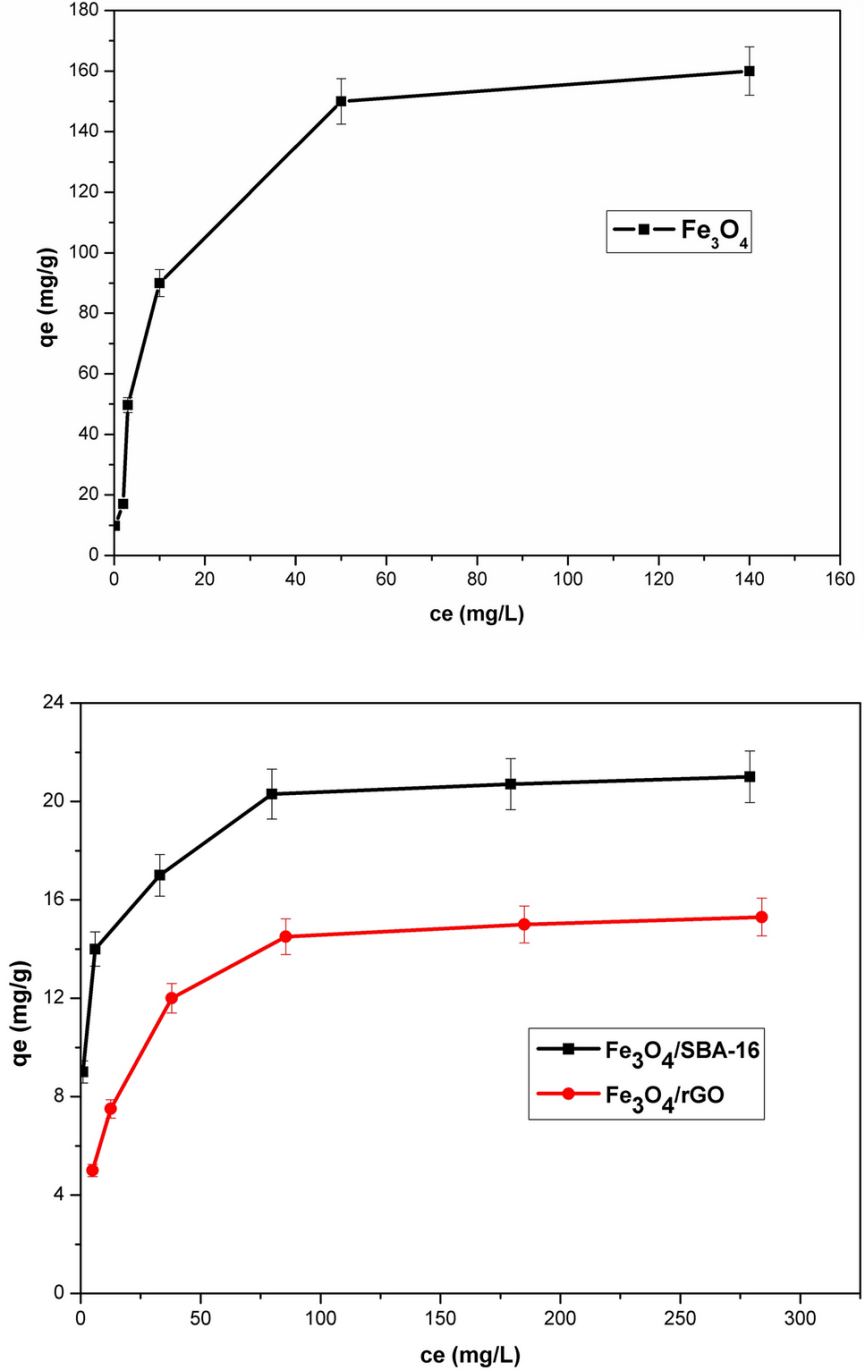
Adsorption isotherm plateau of samples toward AB40 dye (pH = 3, Time = 24 h and T = 25 °C).

**Fig. 10:**
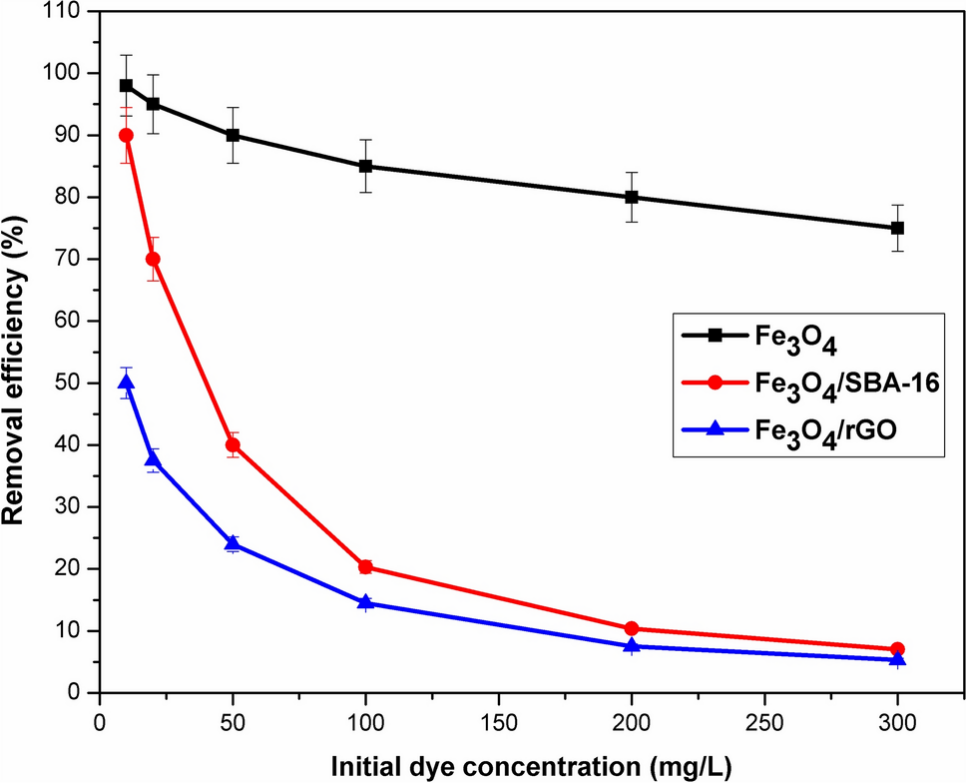
Removal efficiency at different AB40 dye concentrations (Time = 24 h and T = 25 °C).

Figure 9 illustrates the relationship between the equilibrium concentration (C_e_) and the adsorption amount at equilibrium (q_e_) in order to interpret the mechanism of AB40 dye removal. It is evident that the adsorption capacity rises as AB40 dye concentration increases, eventually reaching equilibrium at elevated concentrations. The impact of initial dye concentrations (10–300 mg/L) on the percentage of dye removal at 25 °C by the prepared magnetic catalysts was examined as represented in Fig. 10. Consequently, when the AB40 dye initial concentrations are low, Fe_3_O_4_ exhibits greater removal efficiency compared to other composites. However, with increasing initial concentration, the removal efficiency for AB40 dye by both magnetic composites decreases, but is still high by Fe_3_O_4_. This trend is attributed to the highest surface area and accessible acidic surface sites of Fe_3_O_4_ compared to Fe_3_O_4_/SBA-16 and Fe_3_O_4_/rGO (Fig. 10).

### Langmuir and Freundlich isotherm studies

**Fig. 11:**
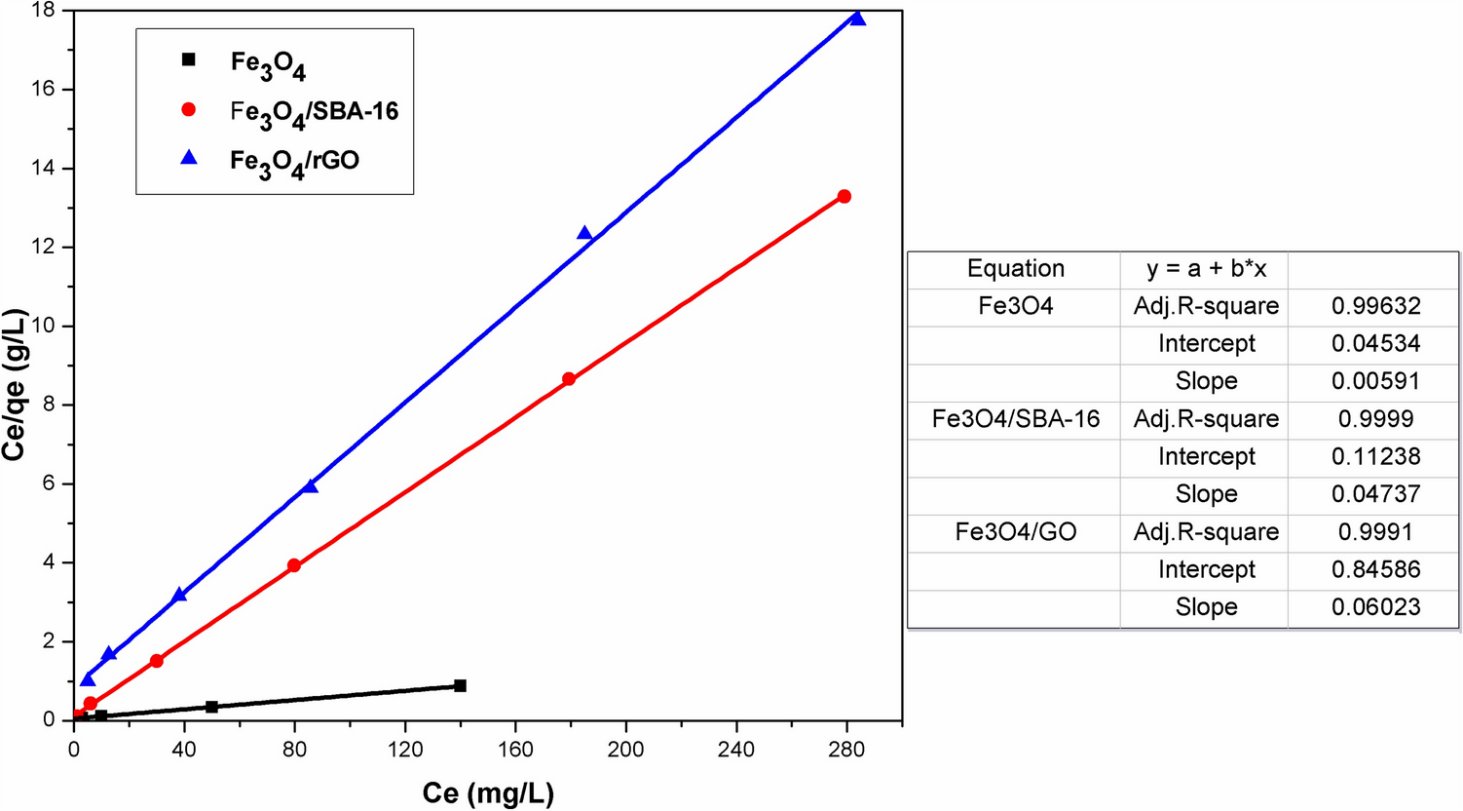
Linear Plot of Langmuir isotherm model for prepared samples.

**Fig. 12:**
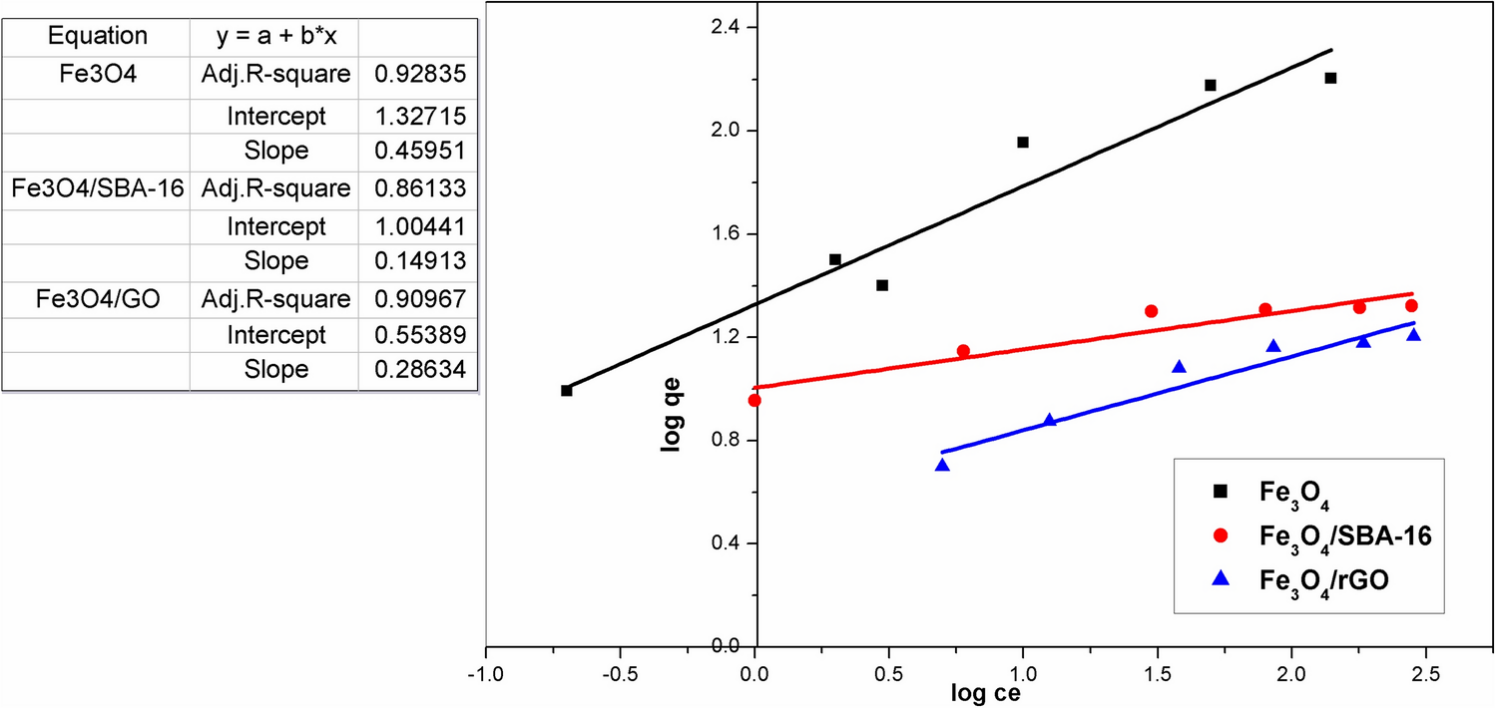
Linear Plot of Freundlich isotherm model for prepared samples.

Langmuir and Freundlich models are widely recognized isotherms used to describe the behavior of dye diffusion between liquid and solid phases^37^ to designate the interaction among the dye solution and produced adsorbents. It can be seen that Figs. 11 and 12 show linear plots of both isotherms for the adsorption of AB40 dye. The calculated parameters and correlation coefficients (*R*^2^) for both adsorption isotherms are summarized in Table 2. The values of R^2^ for the Langmuir isotherm exceeded those of the Freundlich isotherm. This suggests that the Langmuir model is more appropriate for characterizing the adsorption of AB40 dye onto the prepared samples in comparison to the Freundlich model. This model posits that the adsorption process is a physical adsorption that takes place on well-defined specific sites at a finite saturation limit^37,38^. Based on the results deduced from pHpzc and Langmuir adsorption isotherm, therefore, the physical adsorption governs the adsorption mechanism of AB40 dye anions over the prepared magnetic samples. On the other hand, other investigations have reported that the adsorption mechanism of AB40 dye on magnetic surfaces may occur through a chemisorption process, involving the binding of oxygen and nitrogen atoms in the dye molecule with Fe^2+^-based magnetic surfaces^28,30^.

**Table 2:** Parameters of Langmuir and Freundlich models.

Specimens	Langmuir				Freundlich			
	Q_max_ (mg/g)	k_L_ (L/mg)	R_L_	R^2^	k_F_	n	1/n	R^2^
Fe_3_O_4_	169.2	0.13	0.43	0.996	21.24	2.2	0.45	0.928
Fe_3_O_4_/SBA-16	21.1	0.42	0.2	0.999	10.10	6.7	0.15	0.861
Fe_3_O_4_/rGO	16.6	0.07	0.6	0.999	3.6	3.5	0.29	0.909

Table 3 lists a comparison of the maximum adsorption capacities of different adsorbents^27–32^, including the prepared magnetic samples in this study. The calculated maximum adsorption capacity for AB40 dye was higher for Fe_3_O_4_ (169.2 mg/g) than for Fe_3_O_4_/SBA-16 (21.1 mg/g) and Fe_3_O_4_/rGO (16.6 mg/g). From Langmuir isotherm, *R*_*L*_ values were lower than 1, manifesting that the dye was adsorbed favorably. The values of *n* from the Freundlich parameters were greater than unity, implying that the AB40 adsorption process onto the fabricated samples was also effective.

**Table 3:** Reported maximum adsorption capacities (Q_max_, mg/g) in the literature for AB40 dye adsorption and other studied specimens.

Adsorbents	Q_max_ (mg/g)	References
Fe_3_O_4_	169.2	Current study
Fe_3_O_4_/SBA-16	21.1	Current study
Fe_3_O_4_/rGO	16.6	Current study
Fe_3_O_4_	130.5	
Fe_3_O_4_/PANI	216.9	^27^
Granular activated carbon	57.47	^49^
Teak leaf litter powder	80.64	^29^
Activated carbon-Polyaniline Matrix	85.16	^50^
Cone biomass	97.06	^30^
Pine sawdust	133.30	^51^

### Fenton oxidation conditions

#### Significance of pH on dye decolorization

In an acidic environment, the fabricated magnetic catalysts release Fe^2+^ into aqueous solutions^24,38^. These Fe^2+^ ions can activate H_2_O_2_ to generate hydroxyl radicals, as per Fenton’s reaction. Hydroxyl radicals are potent oxidizing entities that decompose a variety of dyes, as shown in the following equations^31^.

10 $${\text{Fe}}^{2 + } + {\text{H}}_{2} {\text{O}}_{2} \to {\text{Fe}}^{3 + } + {\text{OH}}^{ - } + {\text{OH}}^{ \cdot }$$

11 $${\text{OH}}^{ \cdot } + {\text{Dye}} \to {\text{intermediates}} \to {\text{CO}}_{2} + {\text{H}}_{2} {\text{O}}$$

Furthermore, H_2_O_2_ can react with Fe^3+^ linked to the Fe_3_O_4_ surface to produce Fe(II) and O^·2−^ radicals^24^.

**Fig. 13:**
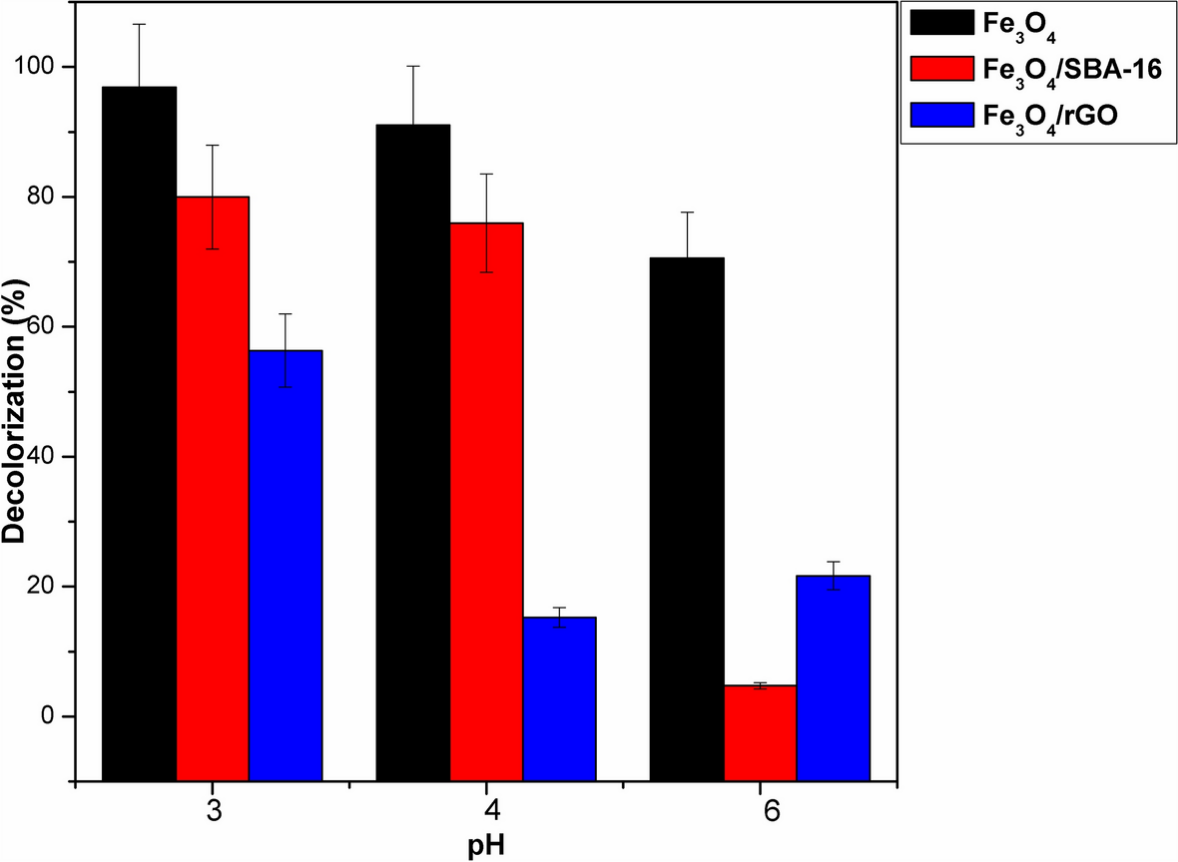
Effect of pH on decolorization (%) of AB40 dye (catalyst dose = 1 g/L, C_o_ = 50 mg/L, time = 60 min, T = 25 °C, and [H_2_O_2_] = 15 mmol/L).

In this study, the influence of pH between 3 and 6 on the decolorization of AB40 dye over the magnetic samples by the Fenton reaction is clarified in Fig. 13. Results in this figure reveal that the highest rate of AB40 dye degradation is attained at low pH 3 over the three studied samples. This is due to the release of enough HO^·^ radicals and the higher solubility of Fe^2+^ in aqueous solutions^24,39^. By increasing pH, the rate of AB40 dye decolorization decreases sharply. It has been pointed out that Fe^2+^ ions become unstable at high pH levels and transform into Fe^3+^ ions, which have the propensity to produce inactive colloidal ferric hydroxo species [Fe(OH)_3_]^24,39^. These species would reduce the creation of HO^·^ and hinder Fenton oxidation. However, the superior rate of AB40 dye decolorization appeared in our study at an acidic pH of 3 aligns with other research^39–44^.

#### Influence of temperature on dye decolorization

**Fig. 14:**
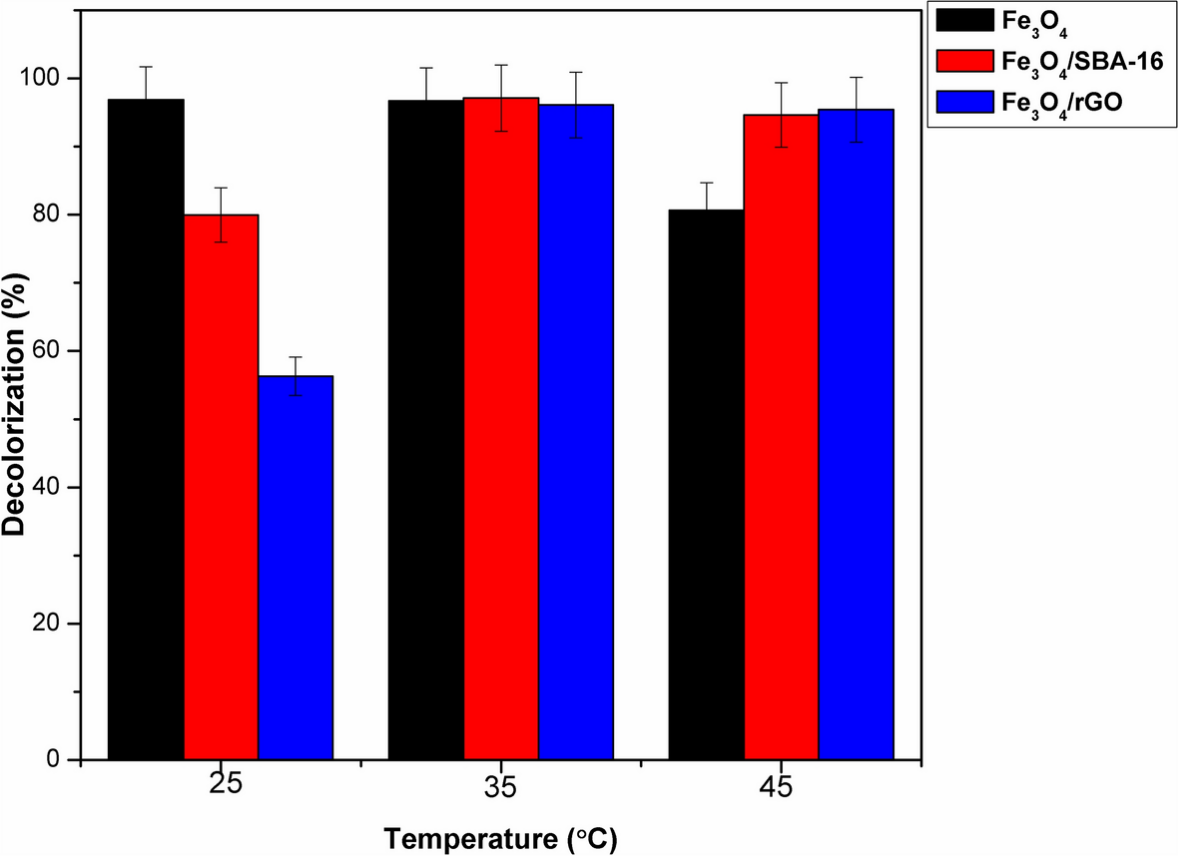
Effect of temperature on decolorization (%) of AB40 dye (catalyst dose = 1 g/L, C_o_ = 50 mg/L, time = 60 min, pH = 3 and [H_2_O_2_] = 15 mmol/L).

The implication of temperature on the decolorization of AB40 dye was examined throughout a range of 25–45 °C. Figure 14 demonstrates that the decolorization efficiency of AB40 dye is significantly influenced by temperature. Increasing the temperature from 25 to 35 °C improved the degradation efficiency of AB40 dye using Fe_3_O_4_/SBA-16 and Fe_3_O_4_/rGO composites, with percentages rising from approximately 79.9 and 56% to around 97.1 and 96.1%, respectively. The reason for this is that higher temperatures can accelerate the reaction rate between H_2_O_2_ and Fe^2+^ ions, thereby increasing the production rate of HO^·^ radicals^44^. However, a further increase in temperature to 45 °C led to a slight decrease in dye decolorization, with percentages dropping from about 97% to 80.6%, 94.6% and 95.4% using Fe_3_O_4_, Fe_3_O_4_/SBA-16, and Fe_3_O_4_/rGO, respectively. It is noteworthy that both composites show higher dye decolorization at elevated temperatures compared to Fe_3_O_4_ alone. This could be interpreted as the stability of Fe_3_O_4_ being minimized by increasing temperature above 25 °C contributing to the decline in decolorization efficiency. The optimal temperature for the degradation of AB40 dye is 25 °C using Fe_3_O_4_ and 35 °C using Fe_3_O_4_/SBA-16 and Fe_3_O_4_/rGO composites. These findings suggest that a synergistic effect is developed when Fe_3_O_4_ supported on SBA-16 and rGO at varying temperatures from 25 to 45 °C and enhanced the stability of such Fenton-like catalysts.

#### Influence of [H_2_O_2_] on decolorization of AB40 dye

**Fig. 15:**
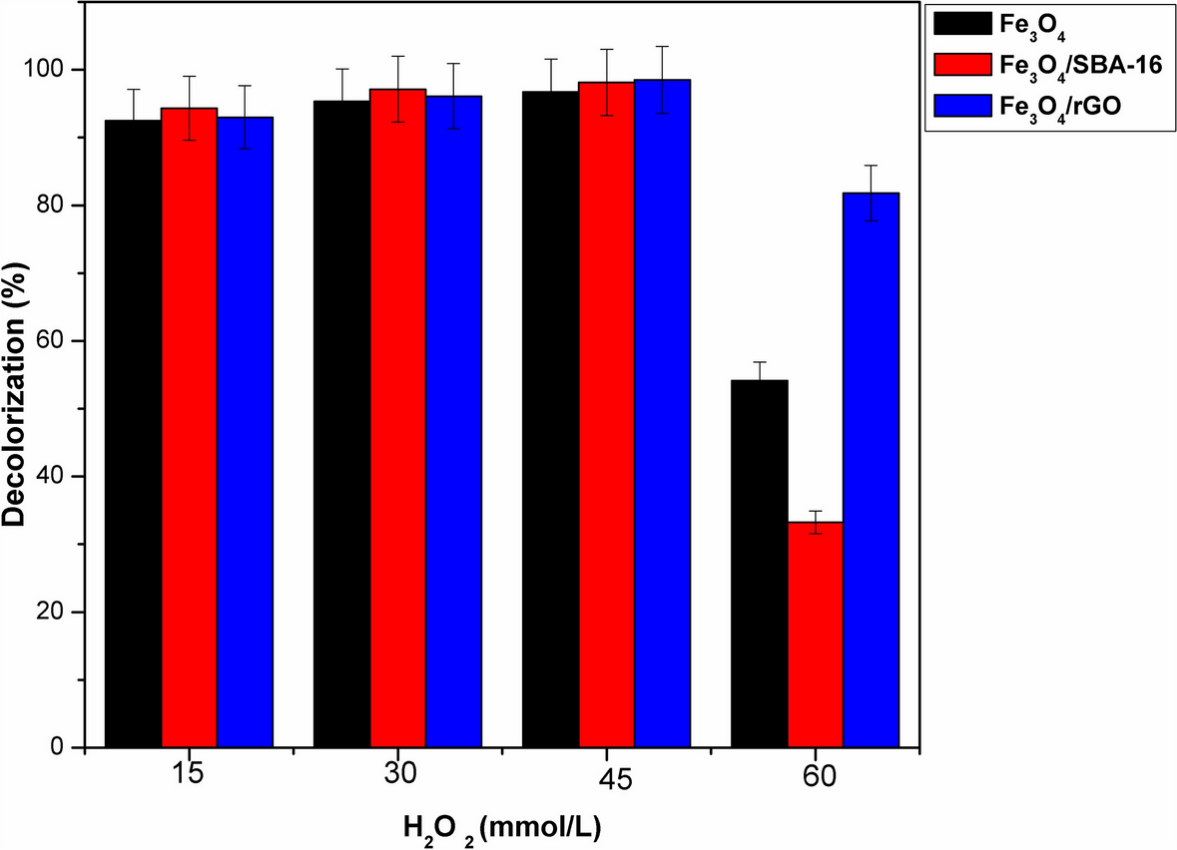
Effect of [H_2_O_2_] on decolorization (%) of AB40 dye (catalyst dose = 1 g/L, C_o_ = 50 mg/L, T = 35 °C, time = 60 min and pH = 3).

To achieve the impact of H_2_O_2_ alone on the decolorization of AB40 dye (C_o_ = 50 mg/L), a blank experiment was performed at [H_2_O_2_] = 45 mmol, pH = 3, and T = 35 °C. Fig. S2 showed that a maximum dye decolorization of roughly 10% at 120 min was obtained with H_2_O_2_ alone. This may be attributed to the weak generation of HO^·^ radicals in the absence of catalysts. On the other hand, the effect of H_2_O_2_ at a range of 15–60 mmol/L with catalysts was examined with a pH of 3 and an initial AB40 dye concentration of 50 mg/L at a temperature of 35 °C. Figure 15 explains that the decolorization of AB40 dye is significantly influenced by the strength of H_2_O_2_. The results showed that the decolorization of the dye varied from 92 to 98% as the concentration of the oxidizing reagent changed from 15 to 45 mmol/L using Fe_3_O_4_, Fe_3_O_4_/SBA-16, and Fe_3_O_4_/rGO, respectively. However, when the concentration was further increased, the dye decolorization dropped dramatically to 54.1, 33.3, and 81.7% using Fe_3_O_4_, Fe_3_O_4_/SBA-16, and Fe_3_O_4_/rGO, respectively. According to Elham et al.^45^, the highest color decolorization efficiency for AB40 dye was achieved at H_2_O_2_ concentration of 45 mmol/L. In summary, an excessive amount of H_2_O_2_ can give rise to the formation of an inert iron oxide coating on the surface of magnetic samples, preventing further Fe^2+^ formation into the solution. Additionally, H_2_O_2_ can scavenge hydroxyl radicals during the process and generate perhydroxyl radicals (HO_2_^·^)^41^, thereby reducing the decolorization efficiency of AB40 dye.

#### Influence of dye concentration on Fenton-like catalysts efficiency

**Fig. 16:**
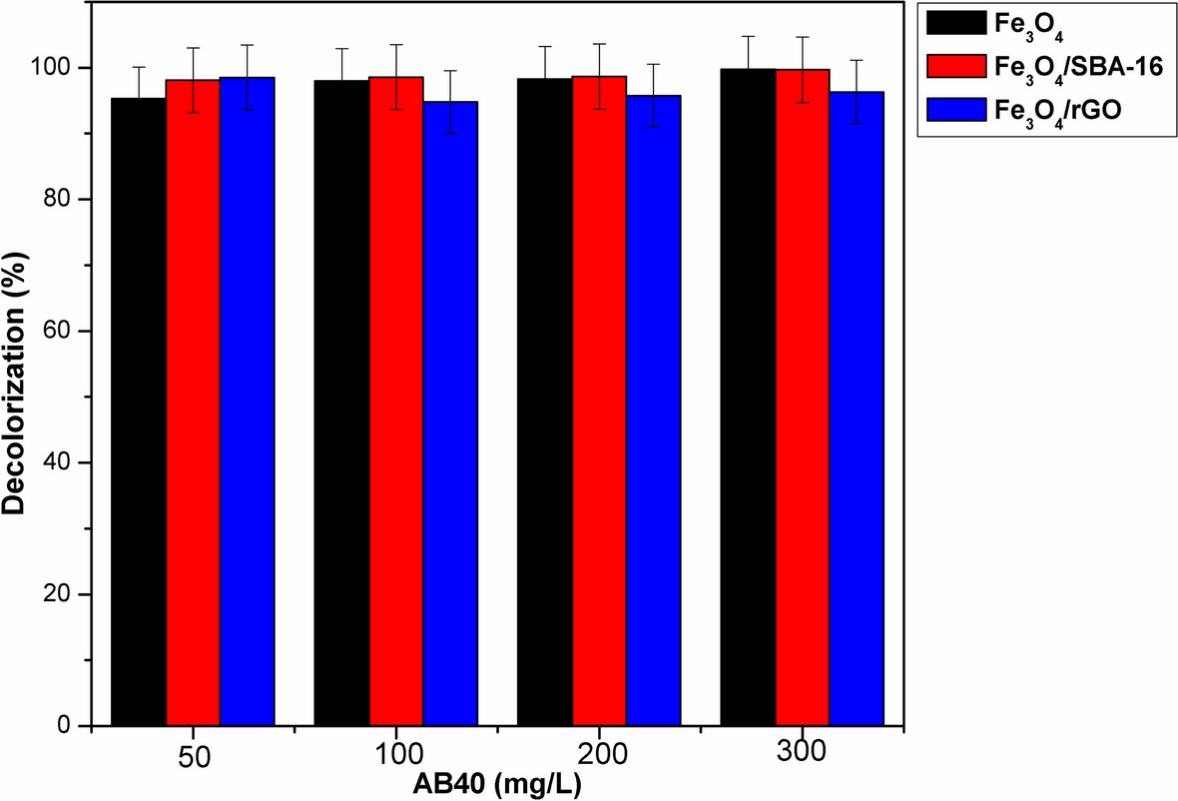
Effect of AB40 concentration on its decolorization (%) (catalyst dose = 1 g/L, [H_2_O_2_] = 45 mmol, pH = 3, time = 60 min and T = 35 °C).

The Fenton catalytic efficiency of magnetic NPs and their composites may be influenced by variations in dye concentration. In order to examine the impact of initial dye concentration on Fenton catalytic performance, different AB40 dye initial concentrations (50, 100, 200 and 300 mg/L) were employed with [H_2_O_2_] = 45 mmol, pH 3, and at 35 °C. Figure 16 illustrates that there is a full decolorization upon increasing AB40 dye concentration from 50 to 300 mg/L using Fe_3_O_4_ and Fe_3_O_4_/SBA-16, except Fe_3_O_4_/rGO showed a slight reduction in dye decolorization (~ 95%). On the other hand, previous reports pointed out that the over concentration of AB40 dye can form two dye radicals upon attacking the hydroxyl radicals, and hence they may dimerize to form a larger molecule. Thus, the catalytic oxidation of the formed dimers is often a challenging to the decolorization process due to its intense color in the solution^26,45^. Accordingly, the optimized Fenton oxidation conditions here could exhibit comparable and strong decolorization of AB40 dye using the prepared magnetic catalysts. This result confirms that the prepared catalysts have superior Fenton activity to work effectively with higher AB40 dye concentrations.

#### Dynamic study of Fenton reaction

To determine the effect of reaction time on the decolorization rate using magnetic samples, experiments were conducted with reaction times ranging from zero to 100 min. The decolorization process of AB40 takes place in two steps. The first step occurs as early as 2 min with fast decolorization rates of 97, 93, and 40% for Fe_3_O_4_, Fe_3_O_4_/SBA-16 and Fe_3_O_4_/rGO, respectively, at an initial dye concentration of 100 mg/L, pH 3 and 35 °C. After that, the decolorization rate slows and attains equilibrium, with a maximum removal of about 99.9% after 25 min for Fe_3_O_4_, 99% after 45 min for Fe_3_O_4_/SBA-16, and 63% after 100 min for Fe_3_O_4_/rGO, respectively.

**Fig. 17:**
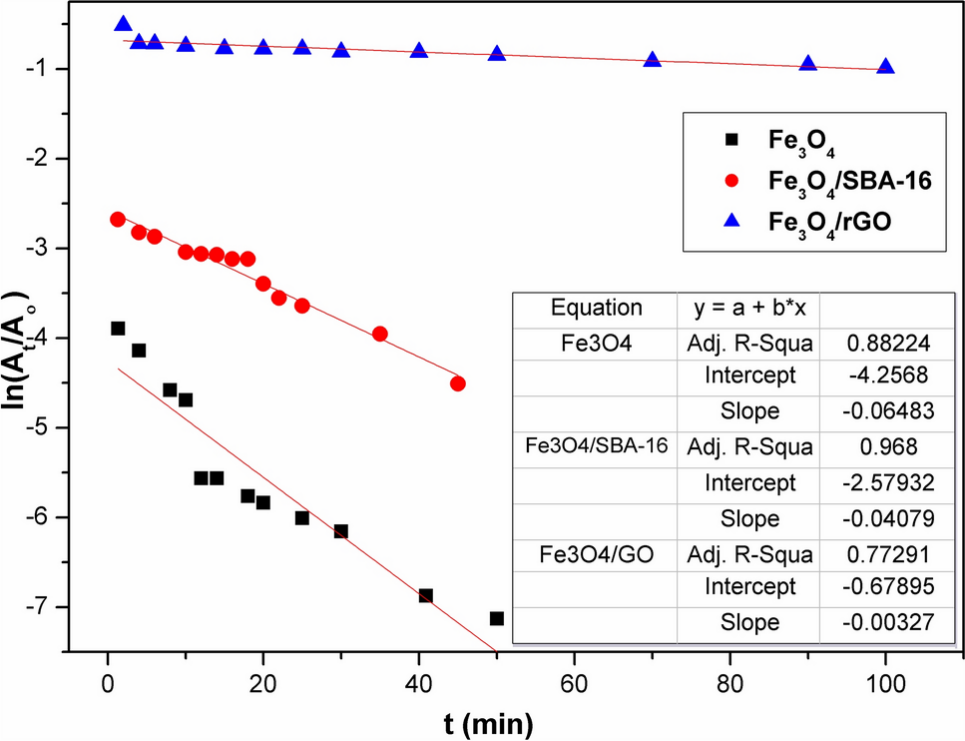
Plot of first-order kinetic model for AB40 dye degradation.

**Fig. 18:**
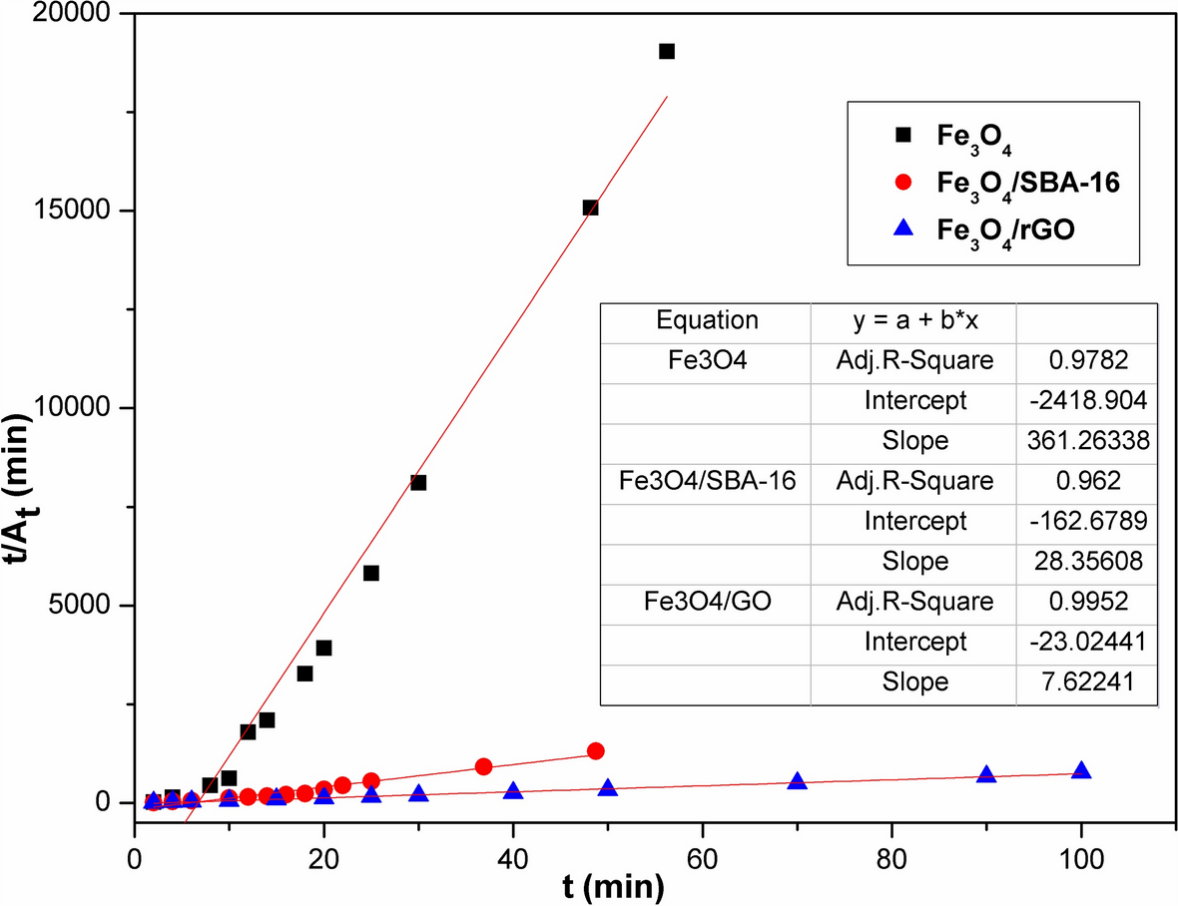
Plot of second-order kinetic model for AB40 dye degradation.

The decolorization of AB40 dye by Fenton–like catalysts applying linear regressions of first- and second-order kinetic models was studied. The outcomes of k_1_ and k_2_ for these processes have been calculated, and the findings are displayed in Figs. 17 and 18 and Table 4. It can be seen that second-order reaction kinetics (R^2^ > 0.95) appeared to be the most suitable model for capturing Fe^2+^-containing oxidation reactions, accompanied by first-order-kinetics^3,46^. The pseudo-second-order rate (k_2_, min^−1^) constants for Fe_3_O_4_-based composites are significantly affected by the insertion of SBA-16 and rGO, dropping from − 45.9 min^−1^ using Fe_3_O_4_ to − 5.2, and − 2.6 min^−1^ for Fe_3_O_4_/SBA-16 and Fe_3_O_4_/rGO, respectively^45^. Fenton reactions, which use Fe^2+^ as a catalyst, often oxidize organic molecules in two stages: a rapid one and a considerably slower one. The faster stage is ascribed to an interaction between Fe^2+^ and H_2_O_2_, whereas the slower stage is caused by the buildup of Fe^3+^ and a limited restoration of Fe^2+^ by H_2_O_2_^44–50^. Table 5 presents a comparison of adsorption and Fenton oxidation methods for the degradation of AB40 dye. Adsorption of AB40 dye by the samples is much lower than that by Fenton oxidation. The decolorization efficiency of AB40 is enhanced greatly and faster when Fenton-like operations are applied using the prepared catalysts in the following order: Fe_3_O_4_ = Fe_3_O_4_/SBA-16 > Fe_3_O_4_/rGO.

**Table 4:** Kinetic parameters of Fenton test for prepared catalysts.

Parameters	Fe_3_O_4_	Fe_3_O_4_/SBA-16	Fe_3_O_4/_/rGO
Pseudo 1st order			
K_1_ (min^−1^)	0.065	0.041	0.003
R^2^	0.88224	0.968	0.773
Pseudo 2nd order			
K_2_ (min^−1^)	− 45.9	− 5.2	− 2.6
R^2^	0.9782	0.962	0.9952

**Table 5:** Percentage removal efficiency of AB40 dye (C_o_ = 100 mg/L) by adsorption and oxidation processes.

	Time	Fe_3_O_4_	Fe_3_O_4_/SBA-16	Fe_3_O_4_/rGO
		% Removal of AB40 dye		
Adsorption process	24 h	85	20.3	14.5
Fenton-like process	60 min	98	98.6	94.8

### Regeneration Study

**Fig. 19:**
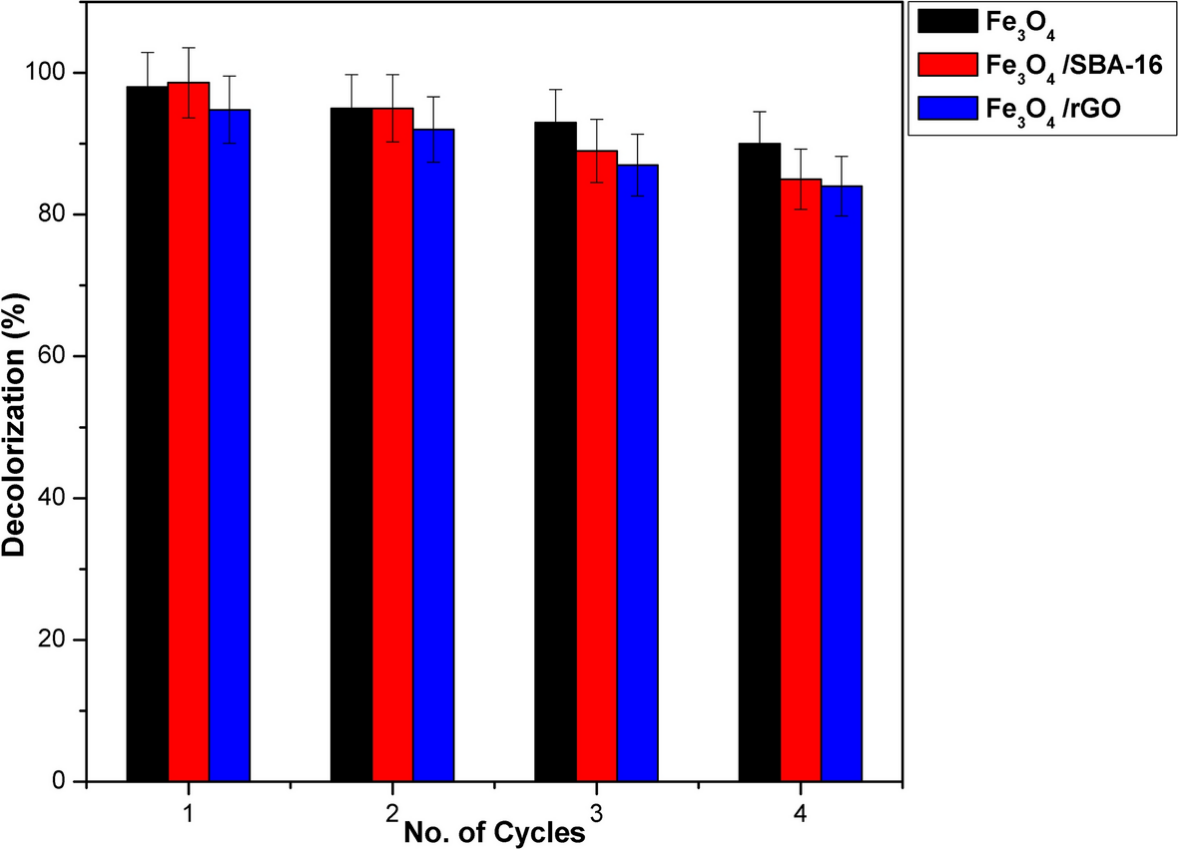
Reusability of samples for degradation of AB40 dye (catalyst dose = 1 g/L, C_o_ = 100 mg/L, pH = 3, T = 35 °C, time = 60 min and [H_2_O_2_] = 45 mmol/L).

The reusability tests conducted on the magnetic catalysts are crucial aspects for economically viable water treatment. The previously fabricated magnetite and catalysts underwent reactivation through repeated washing in double-distilled water. Subsequently, the final product was dried at 80 °C and subjected to a further Fenton decolorization of AB40 dye (catalyst dose = 1 g/L, C_o_ = 100 mg/L, T = 35 °C, [H_2_O_2_] = 45 mmol/L, and time = 60 min) up to four subsequent cycles. The outcomes of dye decolorization are illustrated in Fig. 19 and Fig. S3. After four full cycles under optimal conditions, the regeneration rate attains about 90, 85 and 84% for Fe_3_O_4_, Fe_3_O_4_/SBA-16_,_ and Fe_3_O_4_/rGO, respectively. According to previous studies^50–52^, the reduction in efficiency can be owed to can be owed to the fall in the catalyst mass during the recycling experiments. Moreover, the decolorization rate of the Fe_3_O_4_ sample was better than that of its composites even after the fourth cycle. This is attributed to its larger specific surface area and Fe^2+^ binding active sites (Table 1). However, it can be disclosed that the utilized Fe_3_O_4_-based catalysts may be efficiently regenerated and recycled with little loss of their decolorization efficiency due to pore blocking and disappearing of some active sites.

## Conclusions

This work was primarily focused on the preparation of novel magnetic catalysts containing Fe_3_O_4_ supported on SBA-16 and rGO substrates obtained from rice husk ash in order to investigate their removal performance toward AB40 dye during both adsorption and Fenton-like oxidation processes. FE-SEM, EDX, TEM, FTIR, XRD and nitrogen adsorption measurements affirmed the successful preparation of Fe_3_O_4_, Fe_3_O_4_/SBA-16 and Fe_3_O_4_/rGO samples. Due to its smaller particle size and large total surface area with more acidic sites, the prepared Fe_3_O_4_ sample showed superior adsorption capacity (169.2 mg/g) and Fenton-like oxidation performance at 25 °C compared to other magnetic catalysts. However, conducting Fenton-like oxidation reactions at 35 and 45 °C exhibited that the performance of Fe_3_O_4_/SBA-16 and Fe_3_O_4_/rGO catalysts significantly increased. This result was attributed to the synergistic effect developed between Fe_3_O_4_ with either SBA-16 or rGO resulting in an enhancement in the stability of Fe^2+^-based catalysts to generate an excess of hydroxyl radicals at elevated temperatures. Under optimal conditions such as a catalyst dose = 1 g/L, C_o_ = 100 mg/L, pH = 3, and T = 35 °C, the regeneration performance of magnetic catalysts showed a great decolorization of AB40 dye throughout carrying out four cycles. These outcomes signify the potential use of such prepared magnetic materials as Fenton-like catalysts for treating dye-containing wastewater.

## Supplementary Information

Supplementary Information.

## Data Availability

The authors declare that the data supporting the findings of this study are available within the paper. All data are available from the corresponding author upon request.
