# Supplementary material for: Acid blue 40 dye decolorization using magnetite nanoparticles with reduced graphene oxide and mesoporous silica as Fenton catalysts
Source: Sci Rep. 2025 Mar 14;15:8798. doi: 10.1038/s41598-025-91382-5 (PMC11909164; doi:10.1038/s41598-025-91382-5)
Supplement: Supplementary file 1 — Supplementary Information. [file 41598_2025_91382_MOESM1_ESM.doc]

**Acid Blue 40 Dye Decolorization Using Magnetite Nanoparticles with Reduced Graphene Oxide and Mesoporous Silica as Fenton Catalysts**

**Nady Fathy^*^, Khadiga Abas, Amina Attia, Mona Shouman**

Physical Chemistry Department, Advanced Materials Technology and Mineral Resources Research Institute, National Research Centre, 33 EL Buhouth St., Dokki, Cairo, P.O. 12622, Egypt.

***Correspondence** E-mail: fathyna.77@hotmail.com, na.fathy@nrc.sci.eg

ORCID: <http://orcid.org/0000-0002-6522-1053>

1. **Optimization of Point of Zero Charge (pH_PZC_)**

To determine pH_PZC_ of each solid sample, the initial pH values (pH_i_) of the dye solutions in presence of solid sample were adjusted by adding 0.1 N HCl and 0.1 N NaOH. The studies were conducted out in 50-mL flasks containing 20 mL of the solution at various initial pH values (2-11), as well as 0.02 g of solid sample, while shaking for 24 hours. Following filtration, each supernatant's final pH (pH_f_) was determined using a pH meter (HANNA® bench meter, HI111). pHpzc was determined using the plateau of the pH_f_ vs pH_i_ curve.


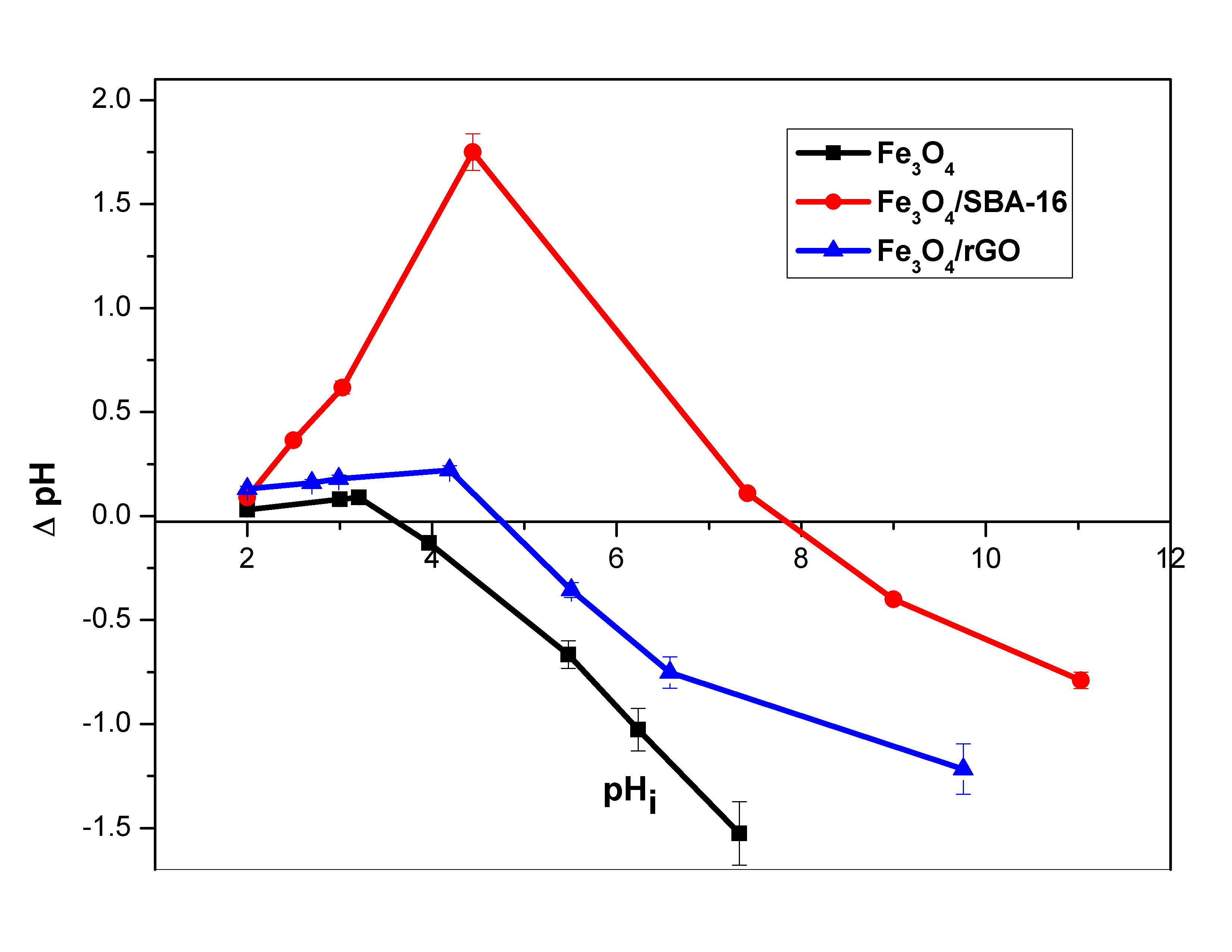


**Fig S1. pH_pzc_ study using pH drift method**

**2. Non catalytic experiment**

To achieve the impact of H_2_O_2_ alone on decolorization of AB40 dye (C_o_= 50mg/L), a blank experiment was performed at [H_2_O_2_] = 45 mmol, pH = 3, and T = 35 °C.

**
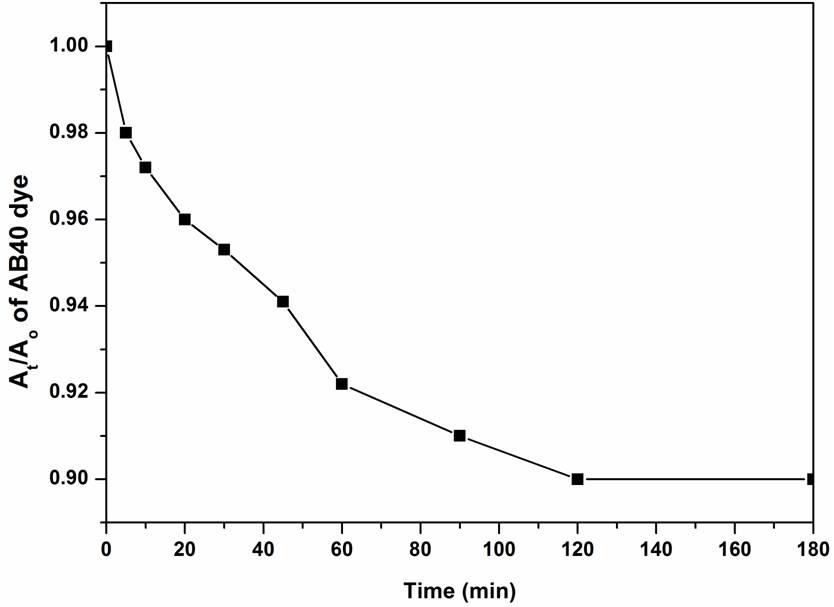
**

**Fig S2.** Impact of H_2_O_2_ on 50 mg/L of AB40 dye decolorization where A° and A_t_ indicate the initial concentration of AB40 and at time (t). ( [H_2_O_2_] = 45 mmol, pH = 3, and T = 35 °C).

**3. UV-Vis Study of Regeneration Cycles**

**
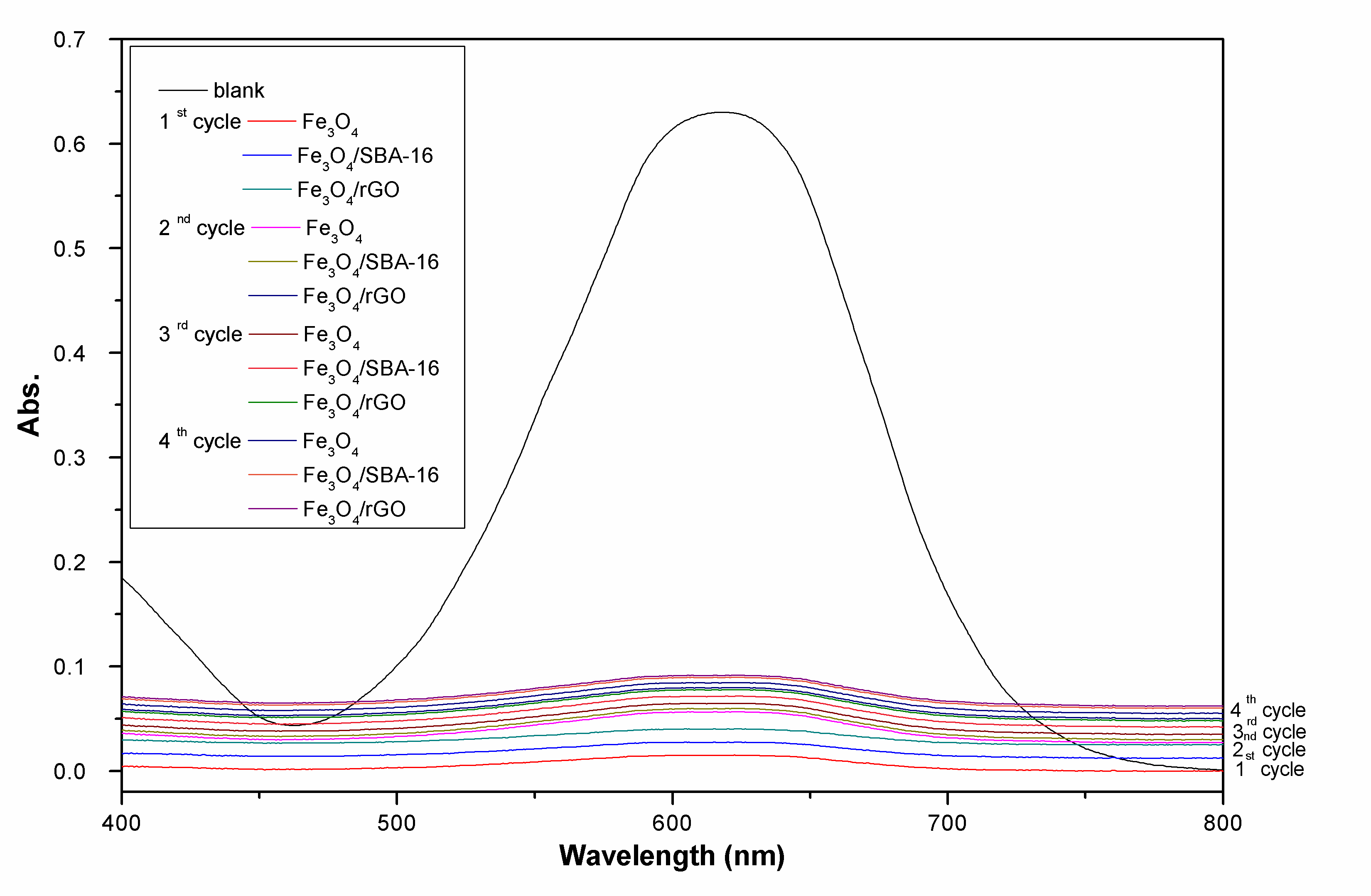
**

**Fig S3. UV-Vis spectra of Regeneration Cycles**
